# Transcriptome analyses reveal genotype- and developmental stage-specific molecular responses to drought and salinity stresses in chickpea

**DOI:** 10.1038/srep19228

**Published:** 2016-01-13

**Authors:** Rohini Garg, Rama Shankar, Bijal Thakkar, Himabindu Kudapa, Lakshmanan Krishnamurthy, Nitin Mantri, Rajeev K. Varshney, Sabhyata Bhatia, Mukesh Jain

**Affiliations:** 1 Functional and Applied Genomics Laboratory, National Institute of Plant Genome Research (NIPGR), Aruna Asaf Ali Marg, New Delhi, India; 2 International Crops Research Institute for the Semi-Arid Tropics (ICRISAT), Patancheru, Telangana, India; 3 School of Applied Sciences, RMIT University, Victoria, Australia; 4 School of Computational & Integrative Sciences, Jawaharlal Nehru University, New Delhi, India

## Abstract

Drought and salinity are the major factors that limit chickpea production worldwide. We performed whole transcriptome analyses of chickpea genotypes to investigate the molecular basis of drought and salinity stress response/adaptation. Phenotypic analyses confirmed the contrasting responses of the chickpea genotypes to drought or salinity stress. RNA-seq of the roots of drought and salinity related genotypes was carried out under control and stress conditions at vegetative and/or reproductive stages. Comparative analysis of the transcriptomes revealed divergent gene expression in the chickpea genotypes at different developmental stages. We identified a total of 4954 and 5545 genes exclusively regulated in drought-tolerant and salinity-tolerant genotypes, respectively. A significant fraction (~47%) of the transcription factor encoding genes showed differential expression under stress. The key enzymes involved in metabolic pathways, such as carbohydrate metabolism, photosynthesis, lipid metabolism, generation of precursor metabolites/energy, protein modification, redox homeostasis and cell wall component biogenesis, were affected by drought and/or salinity stresses. Interestingly, transcript isoforms showed expression specificity across the chickpea genotypes and/or developmental stages as illustrated by the AP2-EREBP family members. Our findings provide insights into the transcriptome dynamics and components of regulatory network associated with drought and salinity stress responses in chickpea.

Chickpea (*Cicer arietinum* L.) is the second most important grain legume and serves as a rich source of proteins (20–25%) and essential amino acids. Chickpea is important for its unique ability to fix atmospheric nitrogen resulting in soil fertility enhancement. The annual total production of chickpea is over 11 million metric tons, of which India alone contributes more than 70%. Although the chickpea production potential is high, it has not been fully realized owing to several abiotic stresses, including drought and salinity stresses^1^,^2^,^3^. More than 40% loss in chickpea yield has been reported worldwide due to terminal drought. The development of stress-tolerant chickpea cultivars is one of the major challenge currently for the researchers. The narrow genetic base in chickpea further limits the efforts to develop stress-tolerant cultivars. The identification of genes associated with drought and salinity stress responses can greatly facilitate the development of improved chickpea cultivars with enhanced drought and/or salinity tolerance using breeding and/or biotechnological approaches.

The availability of large-scale genomic resources is essential for understanding the biology of complex abiotic stresses like drought and salinity. One of the major achievements in this direction is the sequencing of chickpea genome and transcriptome^4^,^5^,^6^. Several efforts have been made to generate marker resources, linkage and physical maps, and quantitative trait loci in chickpea^5^,^6^,^7^,^8^,^9^,^10^. However, only a few studies have been performed to generate functional genomic resources in chickpea. Although quite a few studies have been conducted to identify the genes involved in drought and/or salinity tolerance in chickpea^11^,^12^,^13^,^14^,^15^,^16^,^17^, they were focused mainly either on a single genotype and/or were limited by throughput. Further, data analysis was not comprehensive due to non-availability of the reference transcriptome/genome sequence. Overall, they failed to provide a genome-level understanding of transcriptional responses under abiotic stresses. The availability of next generation sequencing technologies provides a high-throughput means to study gene expression profiles at the whole genome level^18^,^19^. Recently, we performed a genome-wide identification of stress-responsive genes using RNA-seq in chickpea, but this study also focused on a single genotype^20^. However, it has been realized that comparative differential gene expression analysis between genotypes/cultivars with contrasting response to the stresses can provide a better understanding of the molecular mechanisms underlying tolerance and provide better candidate genes^21^,^22^,^23^,^24^.

In the present study, we performed various phenotypic analyses and deep sequencing of the transcriptomes of drought/salinity tolerant and sensitive chickpea cultivars under control and stress conditions at vegetative and/or reproductive stages of development. The reference-based assembly led to the identification of several novel gene loci and different alternatively spliced transcript isoforms. Several genes exhibiting developmental stage and/or genotype-specific differential stress responses were identified. Gene ontology (GO) enrichment and pathway analysis revealed changes in several biological processes and metabolic pathways in response to drought and salinity stresses. These data can facilitate the deployment of various approaches for generation of stress-tolerant chickpea varieties.

## Results

### Phenotypic responses of chickpea genotypes to drought and salinity stresses

Two well-characterized chickpea genotypes with contrasting response to drought stress (ICC 4958 as drought-tolerant and ICC 1882 as drought-sensitive) and salinity stress (JG 62 as salinity-tolerant and ICCV 2 as salinity-sensitive) were selected, which have been used extensively for generation of biparental mapping populations and quantitative trait locus mapping^9^,^16^,^25^,^26^.

The two drought-related genotypes, ICC 4958 and ICC 1882, exhibited differences in the phenology at early reproductive (ER) and late reproductive (LR) development stages. We estimated various phenotypic parameters to confirm their differential stress response at both ER and LR stages. At ER stage, ICC 4958 produced greater root (0.05 g more) and shoot (2.14 g more) biomass, and substantially larger (17%) roots as compared to ICC 1882 under drought stress (Table 1). The roots were thinner (0.05 mm) in ICC 4958 as compared to ICC 1882. The specific leaf area (SLA) of ICC 4958 was comparatively lesser (39 cm^2^ g^−1^) under drought stress. The chlorophyll content and relative water content (RWC) were similar in both the genotypes under drought stress. At LR stage, the shoot biomass was higher (3.3 g) in ICC 4958 as compared to ICC 1882 under stress, whereas the root biomass was similar (Table 1). The total root length was reduced significantly in ICC 4958, whereas significantly thinner roots helped to grow them longer in ICC 1882. Further, SLA was significantly higher (59 cm^2^ g^−1^) in ICC 4958 as compared to ICC 1882. The chlorophyll content was slightly higher in ICC 1882 and RWC was similar in both the genotypes at the LR stage (Table 1).

The genotypes used for salinity stress (JG 62 and ICCV 2) also showed phenological differences under control conditions. In the field trials, it has been observed that ICCV 2 flowered in 35–37 days and JG 62 flowered 53–54 days after sowing. Both the genotypes produced similar shoot biomass at crop maturity under salinity stress, whereas the grain yield of JG 62 was about two times higher than that of ICCV 2. We estimated various phenotypic parameters of these genotypes to confirm their differential salinity stress response at vegetative (Veg) and LR stages. At the Veg stage, JG 62 produced 20% lesser shoot biomass than ICCV 2, whereas the root dry weight, total root length and average root diameter were similar across the genotypes under salinity stress (Table 2). SLA was more by 34 cm^2^ g^−1^ in ICCV 2 compared to JG 62 under salinity stress. The chlorophyll content and RWC did not vary significantly between the genotypes (Table 2). At the LR stage, the shoot biomass of both the genotypes was similar. However, the root biomass and length decreased significantly in ICCV 2 as compared to JG 62. SLA was more by 56 cm^2^ g^−1^ in JG 62 as compared to ICCV 2. The chlorophyll content and the RWC did not vary significantly between the genotypes. The differences between the genotypes in biomass productivity or any other trait were very minimal at the Veg stage and did not explain better salt tolerance of JG 62. However, this tolerance is well known to be due to the reproductive success or success in producing larger number of seeds^25^.

### Transcriptome sequencing and reference-guided assembly

**Figure 1:**
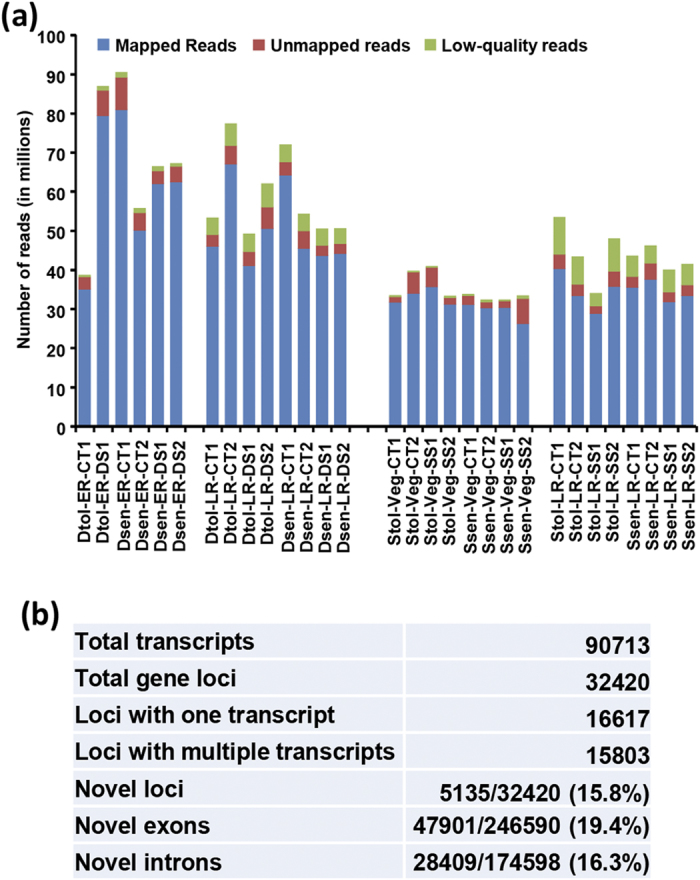
Read mapping and summary of reference-guided assembly. (**a**) Bar graph shows number of low-quality reads removed, mapped reads on the chickpea genome and unmapped reads for each sample. Dtol, drought-tolerant; Dsen, drought-sensitive; Stol, salinity-tolerant; Ssen, salinity-sensitive; ER, early reproductive, LR, late reproductive, Veg, vegetative; CT, control; DS, drought stress; SS, salinity stress. (**b**) Summary of reference-guided assembly and its comparison with the available genome annotation. Total number of transcripts generated, transcript isoforms and novel gene loci/exons/introns identified have been shown.

Root being the first organ exposed to drought and/or salinity stresses, was used for transcriptome analyses. The mock-treated (control) and drought stressed root tissues from the ER and LR stages of the ICC 4958 and ICC 1882 chickpea genotypes were used for RNA sequencing. Likewise, mock-treated (control) and salinity stressed root tissues from the Veg and LR stages of the JG 62 and ICCV 2 chickpea genotypes were used for sequencing. In total, we obtained more than 1.5 billion reads from the 30 tissue samples (minimum 30 million reads for each sample), representing 16 different genotypes/conditions/developmental stages (two biological replicates each of two genotypes under control and stress conditions at two developmental stages for both drought and salinity stresses except for two samples for which enough high-quality data was available from only one replicate). Among these, more than 1.4 billion (93.4%) high-quality reads filtered via NGS QC Toolkit were retained. The high-quality reads were mapped on the kabuli chickpea genome sequence using TopHat2 software. Overall, about 92% (ranging from 80–95% for individual sample) of the high-quality reads mapped to the chickpea (kabuli) genome (Fig. 1a). The mapped read files were used for reference-guided assembly and differential gene expression analysis. For convenience, the phenotype towards drought/salinity stress of the genotype (tolerant/sensitive) along with developmental stage (Veg/ER/LR) and condition (control/drought/salinity) has been used as the sample name (for example, Dtol-ER-CT and Dtol-LR-DS refer to the drought-tolerant-early reproductive-control and drought-tolerant-late reproductive-drought stress, respectively). The summary of sequence data generated, filtered reads and reads mapped on the genome is given in Supplementary Table S1.

We performed a reference-guided assembly of the whole dataset using Cufflinks-Cuffmerge pipeline. This assembly generated a total of 90713 transcripts representing 32420 gene loci. This number is significantly higher than the number of genes annotated in the chickpea genome^6^. This may be due to the availability of incomplete (~74%) chickpea genome sequence as of now. A comparison of the transcriptome assembly in this study with the chickpea genome annotation led to the identification of 5135 (15.8%) novel loci (Fig. 1b). Overall, more than 19% and 16% of exons and introns, respectively, represented in the transcriptome, were novel (Fig. 1b). A putative function could be assigned to 3589 novel transcripts via BLAST search in various protein/nucleotide databases, including TAIR9, Uniref90, Uniref100, nr, Pfam and SMART, whereas the function of others remain unknown. Overall, these results demonstrate the potential of RNA-seq in discovery of novel genes/transcripts in the sequenced genomes as well.

### Global gene expression analysis

**Figure 2:**
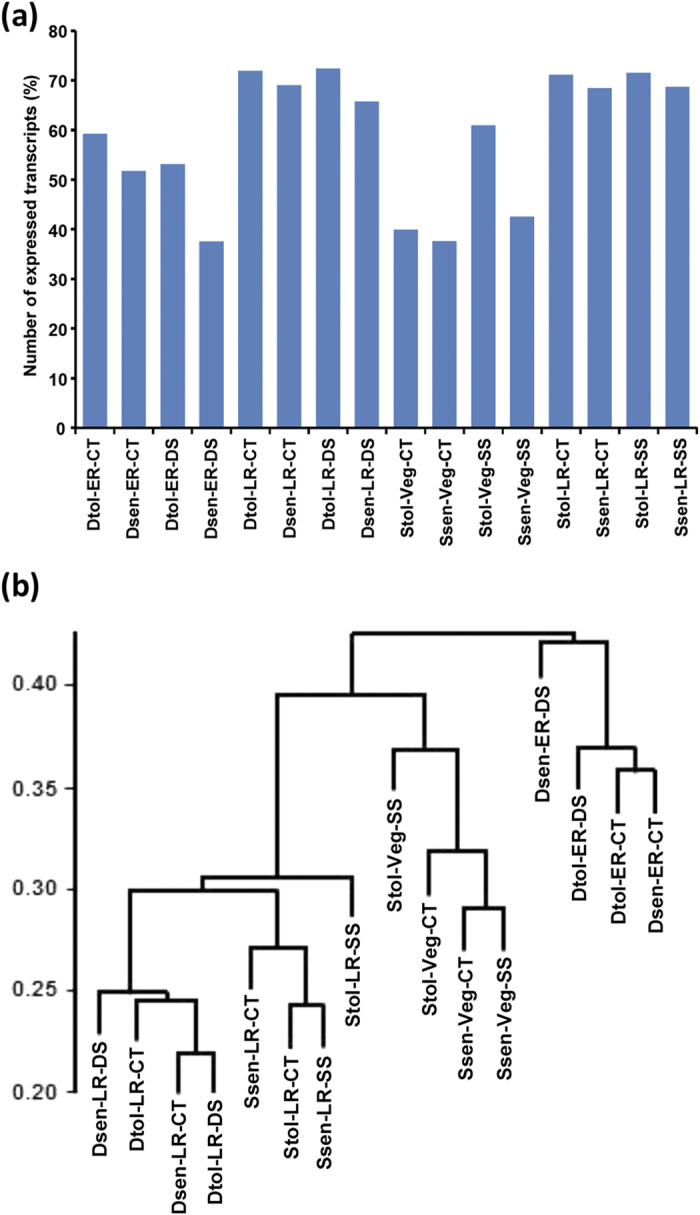
Global gene expression and correlation among the tissue samples. (**a**) Percentage of the transcripts expressed in each sample are shown in the bar graph. (**b**) Dendrogram showing correlation among the different samples based on global expression profiles. The correlation coefficient has been shown with the scale on left side. Dtol, drought-tolerant; Dsen, drought-sensitive; Stol, salinity-tolerant; Ssen, salinity-sensitive; ER, early reproductive, LR, late reproductive, Veg, vegetative; CT, control; DS, drought stress; SS, salinity stress.

The normalized expression level (number of fragments per kilobase of exon per million fragments mapped, FPKM) of each transcript was estimated in all the samples analyzed. A total of 78883 transcripts were identified as expressed in at least one of the 16 samples analyzed. We detected 22,987 transcripts expressed constitutively in all the samples. The number of expressed transcripts varied from 37.5% for Dsen-ER-DS to 72.4% for Dtol-LR-DS sample (Fig. 2a). To investigate the relationship among the transcriptomes of different tissue samples (genotype/developmental stage/condition), we performed a correlation analysis on the normalized expression values from all the samples and generated a dendrogram (Fig. 2b). This analyses revealed that diversity of transcriptome was determined in the order of developmental stage, genotype and experimental condition (Fig. 2b). The transcriptomes of salinity-related genotypes at the Veg stage showed closer correlation. Likewise, the transcriptomes analyzed at the reproductive stages were closer. For instance, the transcriptomes of drought and salinity related cultivars at LR stages were grouped together. Further, at a given developmental stage, the transcriptome of genotypes related to drought/salinity were more related to each other and were clustered together. At ER stage, the transcriptomes of drought-tolerant and sensitive cultivars were more similar under control/stress condition. However, for other three cultivars, higher similarity among the transcriptomes under control and stress conditions of the same genotype was observed. Altogether, our data indicate that different genotypes exhibit divergent gene expression programs at different developmental stages and stress conditions.

### Differential gene expression under drought and salinity stress

**Figure 3:**
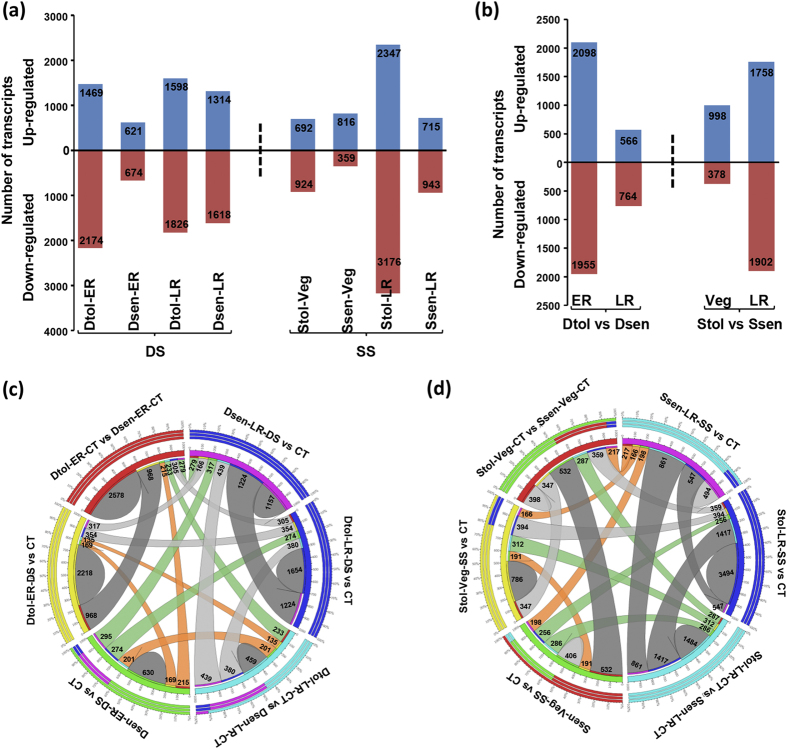
Differential gene expression in the chickpea cultivars under stress and control conditions. (**a**) Number of genes differentially expressed in different chickpea cultivars at vegetative and reproductive stages under drought (DS) or salinity (SS) stress are presented in the bar graph. Number of up- and down-regulated genes are presented via the bars above and below x-axis, respectively. (**b**) Number of genes differentially expressed in tolerant cultivars as compared to sensitive cultivars at vegetative and reproductive stages under control conditions are presented in the bar graph. Number of up- and down-regulated genes are presented via the bars above and below x-axis, respectively. (**c**,**d**) Circos diagram showing overlapping and specific response of differentially expressed genes within drought-related (**c**) and salinity-related (**d**) cultivars under control and stress conditions. The number of transcripts showing specific and overlapping response are given. Dtol, drought-tolerant; Dsen, drought-sensitive; Stol, salinity-tolerant; Ssen, salinity-sensitive; ER, early reproductive, LR, late reproductive, Veg, vegetative; CT, control; DS, drought stress; SS, salinity stress.

To study the differential gene expression, we first filtered out the transcripts with very low expression level in all the samples analyzed. After filtering, we calculated the fold change of each transcript for each genotype and developmental stage under stress condition as compared to the respective control condition and identified the transcripts with significant differential expression (≥two-fold change with P-value ≤ 0.05). Overall, a total of 18462 transcripts representing 13964 unique gene loci exhibited significant differential expression under at least one sample/stress condition. The number of differentially expressed transcripts (DETs) varied from 1295 (for Dsen-ER-DS) to 5523 (for Stol-LR-SS) (Fig. 3a). A larger number of transcripts were downregulated as compared to those upregulated under all the conditions except for Ssen-Veg-SS sample. Under drought stress, larger transcriptional differences between drought-tolerant (3643 DETs) and drought-sensitive (1295 DETs) genotypes were observed at the ER stage. However, a higher extent of transcriptional reprogramming in the salinity-tolerant genotype (5523 DETs) was observed at the LR stage as compared to the sensitive genotype (1658 DETs) under salinity stress. Next, we identified the DETs between the stress-related cultivars under control conditions. A total of 4053 and 1330 genes were differentially expressed at ER and LR stages, respectively, between the two drought-related cultivars (Fig. 3b). Likewise, 1376 and 3660 genes were differentially expressed at Veg and LR stages, respectively, between two salinity-related cultivars. Here also, larger transcriptional differences between drought-tolerant and sensitive genotypes were observed at the ER stage, whereas salinity related cultivars showed greater extent of transcriptional variations at the LR stage.

Further, we analyzed overlap between the conditions in different genotypes at both stages of development. This analysis suggested that a major fraction of DETs were unique to each condition. A total of 7162 (79.1%) transcripts exhibited genotype- and developmental stage-specific differential expression under drought stress (Fig. 3c). Likewise, 7174 (84.8%) transcripts exhibited genotype- and developmental stage-specific differential expression under salinity stress (Fig. 3d). Only a small proportion of transcripts (~20% under drought stress and ~15% under salinity stress) exhibited differential expression in both the genotypes and/or developmental stages. Only 32 transcripts were differentially expressed in both the drought-related genotypes and developmental stages under drought stress. Similar observations were made for the salinity stress, where only 20 transcripts exhibited differential expression in both the salinity-related genotypes and developmental stages. Further, we performed a comparison of differentially expressed unique gene loci under drought/salinity stress in the tolerant/sensitive cultivars with our earlier transcriptome analysis under desiccation and salinity stresses in the roots of ICC 4958 seedlings (Garg *et al.*, 2015). Overall, only a small fraction of genes were found to be common in all the comparisons between the two studies (Supplementary Fig. S1), suggesting the genotype and developmental stage specific stress response in chickpea.

**Figure 4:**
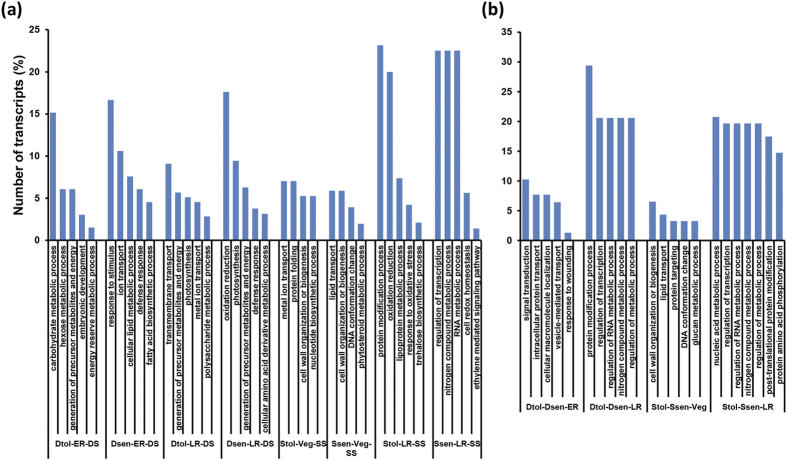
Enriched biological process gene ontology (GO) categories in genes up-regulated in chickpea cultivars under different conditions. The genes up-regulated under stress (a) and control (b) conditions were analyzed using BiNGO and significantly enriched (*P*-value cut-off ≤0.05) biological process terms were extracted. Only few GO categories with highly significant *P*-value represented in the up-regulated genes are shown in the bar graph.

We performed a GO enrichment analysis to assign functional categories to the differentially expressed genes. The genes that encode enzymes involved in various metabolic processes, such as carbohydrate/hexose metabolic processes, lipid metabolic process, energy reserve metabolic process, lipoprotein metabolic process, nitrogen compound metabolic process and oxidation reduction, were found to be greatly enriched under stress conditions (Fig. 4a). The GO terms, cell wall biogenesis, cell redox homeostasis, DNA conformation change and/or ethylene signaling were represented specifically under salinity stress. The genes involved in protein modification process, regulation of transcription and RNA metabolic processes were enriched in the LR stage in drought- and salinity-sensitive cultivars. The GO terms related to transport (ion/metal ion transport and lipid transport) were significantly represented at the ER stage in drought-tolerant and Veg stage in salinity-tolerant cultivar under stress condition. The genes associated with nucleic acid and nitrogen compound metabolic processes, post-translational protein modification and regulation of transcription were significantly enriched among the DETs in drought-tolerant and salinity-tolerant cultivars as compared to the sensitive genotypes under control condition at the LR stage (Fig. 4b). However, the genes involved in signal transduction, intracellular protein transport and vesicle-mediated transport were specifically represented in drought-tolerant genotype at the ER stage. Likewise, genes involved in cell wall organization/biogenesis, lipid transport, protein targeting, DNA conformation change and glucan metabolic processes were significantly enriched in salinity-tolerant cultivar at the Veg stage.

Further, the GO analysis revealed that a large number of transcripts involved in stomata regulation (regulation of stomatal movement/closure), such as those encoding for putative ABC transporter permeases, cytidine deaminases, U-box containing protein, enhancer of mRNA-decapping protein and dihydroflavonol-4-reductase, were found to be differentially expressed in different cultivars and/or stress conditions (Supplementary Fig. S2a). Likewise, a large number of transcripts involved in ion homeostasis (cellular anion/cation homeostasis), such as those encoding for putative glutathione S-transferases, amino acid permeases, phosphatidylinositol-4-phosphate 5-kinases and G3BP-like proteins, were also found to be differentially expressed in different chickpea cultivars and/or stress conditions (Supplementary Fig. S2b).

### Validation of differential gene expression

We performed quantitative reverse transcription (real time) PCR analysis to validate the results of differential gene expression obtained from RNA-seq data. The expression of at least nine genes (selected randomly based on their differential expression patterns under different stress conditions and developmental stages) was validated via RT-qPCR in all the tissue samples. We observed similar gene expression trends (upregulation or downregulation) in RT-qPCR analysis as that of RNA-seq for most of the samples. Further, we determined an overall correlation value of 0.75 (ranged from 0.71 to 0.93 for individual genes) between RNA-seq and qRT-PCR for all (total of 144; average fold change of nine genes in the four genotypes at two developmental stages under stress condition) the data points analyzed (Supplementary Fig. S3). These results suggested a very good agreement between the results obtained via RNA-seq and RT-qPCR.

### Expression trends across the chickpea genotypes, developmental stages and stress conditions

**Figure 5:**
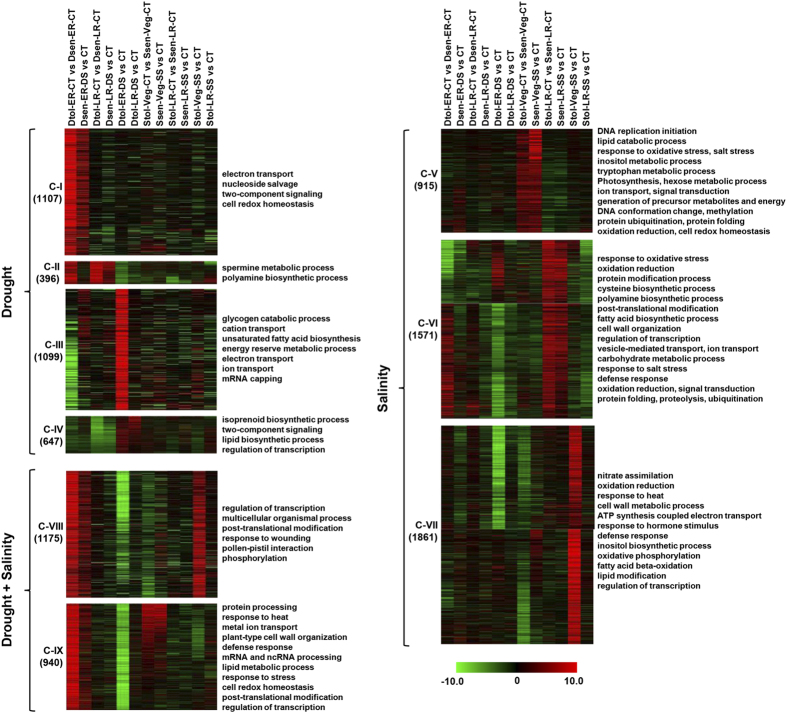
K-means clustering of expression profiles of differentially expressed transcripts under different stress conditions. The clustering was performed on log_2_ fold change for each transcript under different conditions. The transcripts exhibiting similar expression pattern have been grouped together into nine clusters (C-I to C-IX). The number of transcripts included in each cluster are indicated. These clusters were further grouped together based on their specificity of stress response, drought, salinity and drought + salinity. Color scale at the bottom shows log_2_ fold change. The significantly (*P*-value cut-off ≤0.05) enriched biological process GO terms are shown on the right side of each cluster.

To investigate the clusters of genes with similar expression trends across different comparisons, we performed k-means clustering of all the DETs. 11 out of 20 clusters representing 9711 transcripts exhibited unique gene expression profiles across the samples. They contained 396 to 1175 genes. Four of these clusters were merged into two based on their similar expression patterns, thus resulting in a total of nine clusters (C-I to C-IX) (Fig. 5). Each cluster showed distinct characteristics in terms of preferential expression of genes. These clusters were further classified into three superclusters based on the specificity of stress response, including drought, salinity and drought + salinity. Four clusters (C-I to C-IV) of genes exhibited preferential expression in drought-related cultivars, whereas three gene clusters (C-V to C-VII) exhibited preferential expression in salinity-related cultivars. Two clusters, C-VIII and C-IX, exhibited preferential expression in both drought and salinity stress samples. Cluster-I genes (1175) exhibited higher expression in drought-tolerant cultivar as compared to drought-sensitive cultivar at ER stage under control conditions and drought-sensitive cultivar under drought stress. Cluster-VI genes were induced in salinity-tolerant cultivar as compared to salinity-sensitive cultivar at the LR stage under control conditions and salinity-sensitive cultivar under salinity stress.

We assessed each cluster individually for enrichment of biological process GO terms. We observed somewhat distinct and significant functional bias in different clusters (Fig. 5). For example, genes involved in electron transport, nucleoside salvage and cell redox homeostasis were significantly enriched in C-I genes, which were up-regulated in drought-tolerant cultivars under control and drought-sensitive cultivar under drought stress at the ER stage. However, genes involved in spermine and polyamine metabolic processes (C-II) were induced at the LR stage in drought-tolerant cultivars under control condition and in drought-sensitive cultivar under drought stress. The genes involved in glycogen catabolic processes, fatty acid biosynthesis, ion transport and regulation of transcription (C-III and C-IV) were preferentially expressed in drought-tolerant cultivar at ER and LR stages. The genes involved in DNA replication, response to oxidative stress, response to salt stress, photosynthesis, transport, energy generation, cell redox homeostasis, protein folding, regulation of transcription, oxidation reduction, inositol metabolic process etc. were significantly enriched in the clusters (C-V to C-VII) showing response in salinity related cultivars. The cluster of genes showing response in both drought and salinity related cultivars were found to be involved in biological processes, including regulation of transcription, post-translational modification, phosphorylation, ion transport, cell wall organization, mRNA and ncRNA processing, response to stress and cell redox homeostasis etc.

### Differential expression of transcription factor encoding genes

**Figure 6:**
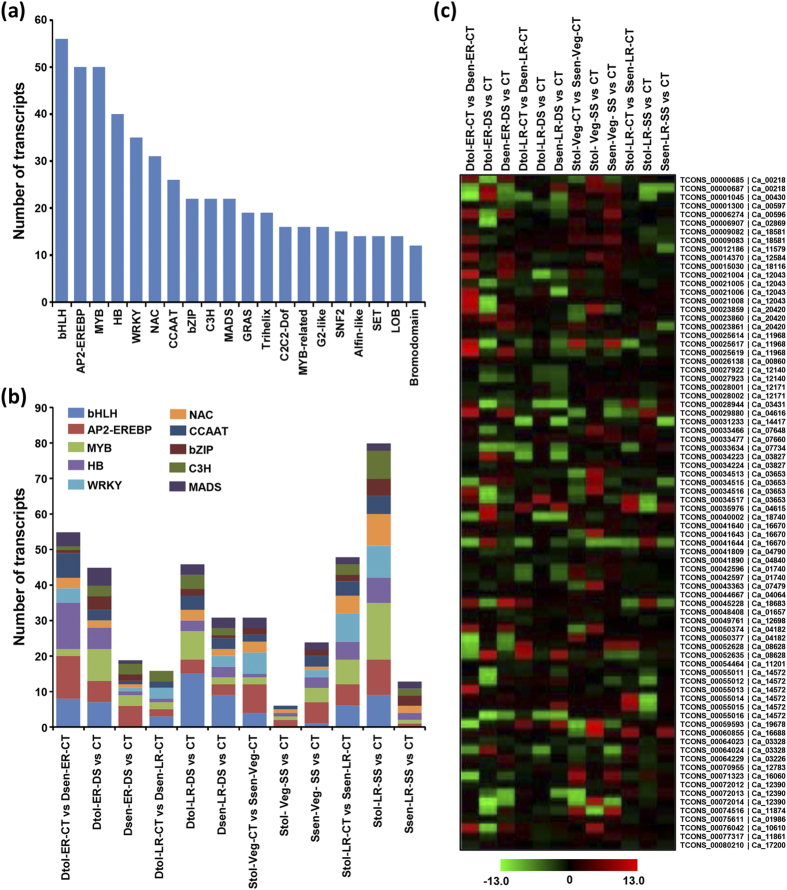
Differential expression of transcription factor (TF) encoding transcripts in chickpea cultivars. (**a**) Top 20 TF families (number of transcripts) represented in the differentially expressed transcripts across all the samples are shown in the bar graph. (**b**) Number of transcripts from top 10 transcription factor families represented in the differentially expressed transcripts showing differential expression in different samples are shown. (**c**) Heatmap showing differential expression of different members (transcript isoforms) of AP2-EREBP TF family. The unique transcript (suffix TCONS) and gene locus identifier for each isoform have been given on right side. The color scale at the bottom represents log_2_ fold change.

We identified the members of 86 transcription factor (TF) families in the chickpea genome, which were represented by 1654 gene loci (5177 transcripts). The differentially expressed TF encoding transcripts were identified by comparisons between control and stress conditions for each genotype at different developmental stages. Among these, at least 775 TF encoding genes (1054 transcripts) belonging to 80 families were differentially expressed under stress conditions. Largest number of members of bHLH gene family were differentially expressed followed by AP2-EREBP and MYB family members (Fig. 6a). A significant number of HB, WRKY and NAC family members also showed response to different stresses. At least 10 members of 24 TF families were found to be differentially expressed. Further, we analyzed the differential expression specificity of top 10 TF families under different stress conditions (Fig. 6b). Although none of TF family could be assigned to a specific stress condition, we observed preferential differential expression of a few TF families under a particular stress condition. For instance, most of the members of bHLH family exhibited differential expression under drought stress (Fig. 6b). Likewise, a larger number of WRKY and NAC TF family members were differentially expressed under salinity stress as compared to drought stress. However, similar number of members of AP2-EREBP and MYB TF families contributed to the drought and salinity stress responses.

Further, we analyzed the differential gene expression of AP2-EREBP TF family members in more detail (Fig. 6c). AP2-EREBP family is represented by at least 146 genes in the chickpea genome. We identified at least 78 transcript isoforms representing 50 unique gene loci to be differentially expressed under the conditions/samples analyzed. Largest number of transcripts were differentially expressed in drought-tolerant cultivar under drought stress at the ER stage (Supplementary Fig. S4). However, a larger number of unique gene loci were differentially expressed in drought-tolerant cultivar at the ER stage as compared to drought-sensitive cultivar. Under salinity stress, a larger number of AP2-EREBP family members were differentially expressed in the salinity-tolerant cultivar at the LR stage (Supplementary Fig. S4). These observations suggest the role of AP2-EREBP family members in drought stress response at the ER stage and salinity stress response at the LR stage. These results are in agreement with that of whole transcriptome level. Further, we observed the genotype, developmental stage and/or stress condition specific differential expression of different isoforms of AP2-EREBP family members (Fig. 6c).

### Regulation of metabolic pathways under stress conditions

**Figure 7:**
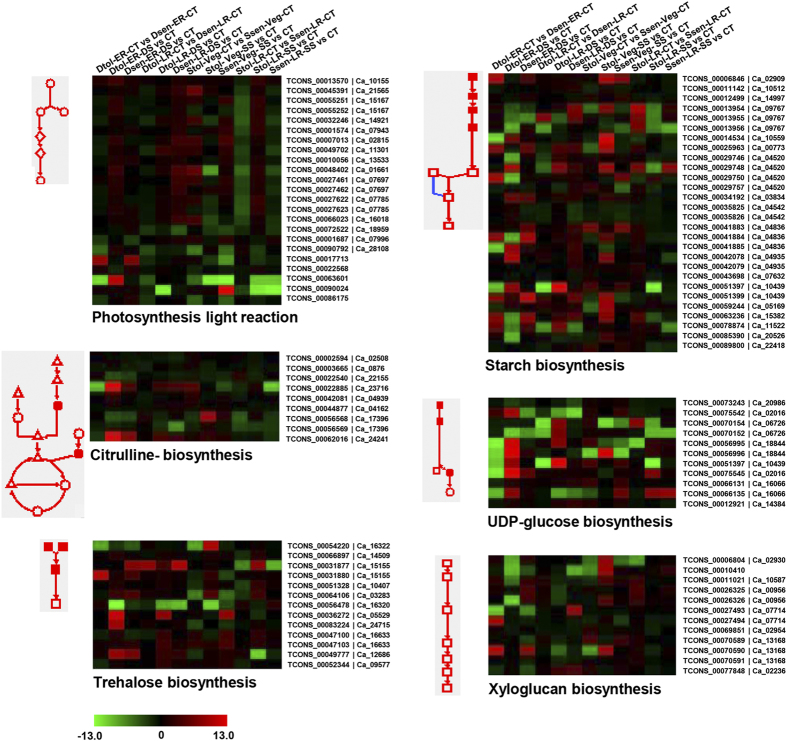
Regulation of metabolic pathways under drought and/or salinity stress conditions. The major metabolic pathways significantly (*P*-value cut-off ≤0.05) enriched in differentially expressed genes are shown. Heatmaps showing the expression profiles of the transcripts involved in these pathways are also shown. The unique transcript (suffix TCONS) and gene locus identifier for each isoform have been given on the right side. The color scale at the bottom represents log_2_ fold change.

We further investigated the possible metabolic pathways involved in drought and/or salinity stress responses using AraCyc database. The enrichment analysis revealed several major metabolic pathways involved in the stress responses. Photosynthesis light reactions was the most significant pathway overrepresented majorly in the salinity stress-responsive genes. The metabolic pathways, UDP-glucose biosynthesis and trehalose (a non-reducing disaccharide) biosynthesis, were significantly represented under drought stress. However, the transcripts involved in starch biosynthesis, citrulline (a non-standard amino acid) biosynthesis and xyloglucan (component of primary cell wall) biosynthesis were significantly enriched in both drought and salinity stress responsive genes. We detected the differential expression of multiple transcript isoforms representing different steps of these metabolic pathways. The heatmaps showing differential expression of the transcripts associated with these pathways are depicted in Fig. 7. In addition, several other metabolic pathways, such as fatty acid degradation, gluconeogenesis, secondary cell wall, proline biosynthesis and generation of precursor metabolites and energy were also significantly represented in the stress-responsive genes. Most of these pathways represented the same biological processes found to be significantly enriched in GO analysis. Further, we performed metabolic pathway analysis of different sets of DETs via MapMan. This analysis also highlighted enrichment of similar pathways, such as those involved in cell wall (trehalose and xyloglucan), photosynthesis, starch/sucrose metabolism, lipid metabolism, and secondary metabolism (phenylpropanoids, terpenes, amino acids and nucleotides), in the DETs in drought and salinity related cultivars (Supplementary Fig. S5, S6). A larger number of DETs involved in various pathways were up-regulated in drought-tolerant cultivars under control and drought stress conditions at ER stage (Supplementary Fig. S5). However, in salinity-tolerant cultivars, a larger number of DETs involved in various pathways exhibited up-regulation under control and salinity stress conditions at LR stage (Supplementary Fig. S6). Overall, these results suggest a crucial role of several metabolic pathways in drought and salinity stress responses.

Several transcripts involved in biotic stress response pathway were also found to be differentially expressed in the chickpea cultivars under control and/or stress conditions. A larger fraction of DETs in the drought-tolerant cultivar at the ER stage under control and drought stress conditions overlapped with the biotic stress response pathways (Supplementary Fig. S7). However, a larger fraction of DETs in the salinity-tolerant cultivar at the LR stage under control and salinity stress conditions overlapped with the biotic stress response pathways (Supplementary Fig. S8). The transcripts implicated in hormone (auxin, brassinosteroid, ethylene, ABA, SA and JA) signaling/metabolism, cell wall, proteolysis, secondary metabolism, redox homeostasis (peroxidases and glutathione-S-transferases), MAPK signaling, defense response and regulation of transcription (ERF, bZIP, WRKY and MYB family TFs) were found regulated under drought and salinity stresses in chickpea. A significant overlap between abiotic and biotic stress responsive genes, suggested a crosstalk between abiotic and biotic stress signaling in chickpea.

## Discussion

The availability of diverse germplasm provide an excellent opportunity to understand the molecular basis of variability in their response to various abiotic stresses, such as drought and salinity. In this study, we analyzed well-characterized chickpea genotypes for their response to drought (ICC 4958 and ICC 1882) or salinity (JG 62 and ICCV 2) stress. Various phenotypic analyses confirmed the contrasting responses of the selected chickpea genotypes to drought or salinity stress. The developmental stage of the genotypes showed confounded effects on stress response^25^,^27^. The drought response had indicated that ICC 4958 had complementary advantages, such as earliness and early growth vigor of both root and shoot systems that confer greater yield under drought stress. At the ER stage, ICC 4958 exhibited thinner roots for better proliferation of root biomass under drought stress, which is an adaptive feature. The reduction of SLA in ICC 4958 was also a similar successful modification in response to drought. At this stage, ICC 4958 has already attained the exponential growth phase, whereas ICC 1882 was way behind. At LR stage, the shoot biomass of ICC 1882 increased as the exponential growth phase was attained. The root length of ICC 4958 reduced substantially at the LR stage as this genotype approached maturity and root death already started. Therefore, the crop growth stage seems to contribute to the drought adaptation. Overall, the phenotypic analyses emphasized the key roles of root system differences and overall phenology in contrasting drought tolerance of the two genotypes^28^.

Previously, JG 62 and ICCV 2 genotypes had been characterized as tolerant and sensitive based on high and low seed yield, respectively, under salinity stress^29^. JG 62 is relatively longer in duration and has the potential to produce two pods per node, thereby partitioning the photo-assimilates in to the grains rapidly^30^. The reduction in shoot biomass at the Veg stage under salinity stress observed here was more related to the phenological differences. More biomass was accumulated in ICCV 2 due to considerable earliness and exponential growth phase. Likewise, root biomass and root length of ICCV 2 reduced substantially under salinity stress at LR stage due to early senescence. Lesser SLA in JG 62 at Veg stage and in ICCV 2 at LR stage under salinity stress can be explained in terms of the growth stage and length of the growth period. It has been shown that the accumulation of sodium continues to occur across the growing period and the concentration of sodium and potassium ions are significantly higher in the sensitive genotypes as compared to the tolerant genotypes^31^. Overall, difference in the salinity tolerance between the two genotypes is not related to their capacity to produce biomass or fill seeds (seed size) under salinity stress, but related to the ability to partition the biomass to the reproductive structures to produce larger number of pods/seeds as shown in previous studies^29^,^32^,^33^.

Understanding the molecular basis of drought/salinity tolerance can facilitate the deployment of genetic engineering and molecular breeding approaches for development of stress-tolerant varieties in crop plants. Although a few genes involved in drought and/or salinity stress response have been identified in chickpea^11^,^12^,^13^,^14^,^15^,^16^,^17^, the molecular mechanism underlying drought/salinity tolerance remains largely unknown. A global transcriptional reprogramming is considered as the important molecular response of the plants to adapt/cope the drought and salinity stress. To understand the molecular response, we performed RNA-seq analysis of the chickpea genotypes (sensitive and tolerant) and investigated transcriptional differences under control and/or stress conditions within/across the genotypes(s). We identified several novel gene loci and transcript isoforms in chickpea, which demonstrated the power of deep sequencing technology. The abundance of thousands of transcripts involved in several biological processes and metabolic pathways was found to be altered in different chickpea genotypes. Most of the transcripts exhibited a genotype and/or developmental stage specific response. Overall analysis of RNA-seq data revealed a complex transcriptional network governing drought and/or salinity stress responses in chickpea.

We observed extensive transcriptional reprogramming in chickpea plants at ER and LR stages under drought and salinity stress, respectively. These results suggested that chickpea plants are more sensitive to drought stress at the ER stage and to salinity stress at the LR stage. Various physiological and phenotypic observations made in previous studies and the present study have demonstrated the higher level of susceptibility of chickpea to various stresses during reproductive development^29^,^31^,^34^. Transcriptome studies conducted in diverse plant species have noted the enrichment of GO terms and metabolic pathways related to stress response^20^,^35^,^36^,^37^,^38^,^39^. We also found the differential accumulation of the transcripts encoding enzymes involved in similar biological processes and metabolic pathways under drought and/or salinity stress conditions in chickpea. For instance, the transcripts involved in various metabolic pathways, cell wall biogenesis, stomatal regulation, ion homeostasis, protein modification process and regulation of transcription were significantly differentially expressed in different chickpea cultivars under stress conditions. Further, our analyses revealed a significant overlap between abiotic and biotic stress responsive genes and several pathways, suggesting a crosstalk between abiotic and biotic stress signaling in chickpea^40^.

The genes encoding enzymes involved in biosynthesis of amino acids (proline and citrulline), polyamines and sugar alcohols (inositol and trehalose) were found to be up-regulated under stress conditions. The production of these osmolytes under abiotic stresses is considered as an adaptive feature^41^. Recently, citrulline has been established as an important biochemical indicator of drought and salinity tolerance^42^. Trehalose is a non-reducing disaccharide present in very low quantity in the plants. The role of trehalose precursor, trehalose-6-phosphate (T6P) as a key regulatory molecule in sugar metabolism, abscisic acid signaling, stress responses and enhancing photosynthetic activity has been established^43^,^44^,^45^. Recently, it has been proposed that fine-tuning of trehalose metabolism can produce stress-tolerant plants without any side-effects^46^,^47^. In addition, we observed that a significantly large number of genes involved in photosynthesis, starch biosynthesis, xyloglucan biosynthesis and UDP-glucose biosynthesis were induced under drought/salinity stress conditions in chickpea. Photosynthesis is a biological process, which needs a tight control under stress conditions. Several studies have reported the regulation of photosynthesis related genes under various abiotic stress conditions^20^,^36^,^48^. Plant hormones, sugars, reactive oxygen species, TFs and protein kinases have been found to regulate the photosynthetic machinery and associated metabolic pathways under abiotic stresses^48^,^49^,^50^. Starch biosynthesis can act as buffer for maintaining optimal status of carbon and energy in the plants under abiotic stress conditions. Accumulating evidences suggest that xyloglucan and UDP-glucose biosynthesis are required for mechanical strengthening and remodeling of cell wall to protect the plants from abiotic stresses^51^. The role of xyloglucan in maintaining root growth has also been proposed, which may provide stress tolerance to the plants^52^,^53^.

In our dataset, about 47% of TF encoding genes were differentially expressed under stress conditions. It has been previously reported that TFs of various families perform a crucial function in abiotic stress responses via gene regulatory networks^20^,^36^,^54^,^55^,^56^. The members of several well-known TF families implicated in abiotic stress responses, such as AP2-EREBP, MYB, NAC and WRKY, were significantly represented among the differentially expressed genes in chickpea. The TF families involved in hormone signaling, such as abscisic acid (ABI3VP1), auxin (Aux/IAA and ARF), gibberellin (GRAS) and cytokinin (ARR) signaling, were differentially expressed, suggesting an important role of plant hormones in drought and salinity stress responses. The role of plant hormones, especially abscisic acid and auxin in abiotic stress responses has been well demonstrated^57^,^58^,^59^,^60^,^61^. The TFs involved in various developmental processes, such as homeobox, MADS-box, ARF and TCP were also found up/down regulated under stress conditions, suggesting their role in developmental stage-specific regulation of stress responses. The role of homeobox TF family in regulation of stage-specific abiotic stress responses has been suggested in previous studies too^62^,^63^. Further, differential expression of multiple isoforms of TFs under different stress conditions can enhance the diversity of their targets. Overall, these results suggest the involvement of a complex transcriptional regulation of various pathways in drought and salinity stress responses. The genes differentially expressed specifically in the tolerant cultivars belonging to different metabolic pathways and transcriptional regulation are the good candidates for further functional analyses. However, the integration of these transcriptome data with other omics and genetics data can help in further selection and pin down the important candidate genes for functional analysis.

In summary, this study provides comprehensive data on differential gene expression in chickpea genotypes with contrasting drought or salinity stress tolerance phenotype. The differences in gene expression between the genotypes at different developmental stages appear to affect the transcription more than the stress condition. A genotype-specific response to drought or salinity stress was more prevalent than the response common to both the tolerant and sensitive genotypes. Further, a different set of genes were found to participate in stress response at vegetative and/or reproductive developmental stages. The genes with dynamic regulation under stress conditions belonged to diverse pathways, mainly metabolic processes, regulation of transcription, protein modification processes and signal transduction. Many of the genes were found to be implicated in biotic stress related pathways as well, which can provide molecular insights into crosstalk between abiotic and biotic signaling. A better understanding of the regulatory function of various components, such as phytohormones, TFs and protein kinases are required to generate abiotic stress tolerant plants. These dataset can be used as starting point to dissect the gene regulatory network involved in drought and/or salinity stress response.

## Methods

### Plant material and stress treatments

Two chickpea genotypes with contrasting phenotype for drought (ICC 4958, drought-tolerant and ICC 1882, drought-sensitive) and salinity (JG 62, salinity-tolerant and ICCV 2, salinity-sensitive) stresses were used in this study. For all the experiments and stress treatments, chickpea plants were grown in a glasshouse/greenhouse (ICRISAT, Hyderabad, India) maintained at a maximum temperature of 25–28 °C and a minimum of 12–22 °C, and a day-time relative humidity of 30–70% with a maximum solar radiation incidence of >1200 *μE* *m*^*−*2^ *s*^*−*1^. Plants were grown in 0.21 m deep pots with 0.25 m diameter containing 9.5 kg of Vertisol soil (pH 8.1, CEC/clay ratio = 0.87, electric conductivity = 0.10 mmhos cm^−1^), fertilized with sterilized farm yard manure as one part for 20 parts soil (v/v) and di-ammonium phosphate at a rate of 300 mg kg^−1^ soil. Three seeds were sown in each pot and maintained under the above mentioned conditions. After 10 days of germination, only one heathy plant per pot was retained for further growth and treatments.

For imposition of drought stress, two sets of ICC 4958 and ICC 1882 genotypes were grown for collecting tissues at early (flowering) and late (podding) reproductive stages. Drought stress was imposed and monitored using a dry down approach^64^. For control plants, pots were maintained at optimum water levels with 90% of the available soil water fraction (ASWF) by irrigating on alternate days. For drought stress, potted soil was maintained at 0.9 ASWF till the start of drought imposition. At the intended time of drought imposition, the pots were irrigated four times with one liter of water each time so as to bring the soil to field capacity. The pots were allowed to drain excess water overnight and weighed. After one day, the surface of each pot was covered with a polythene sheet. The weight of each pot was recorded periodically to monitor the water loss. The roots of the plants were harvested in at least three biological replicates, when ASWF reached to 0.2 at 50 days (ER) and 70 days (LR) for RNA extraction. The control plants were also harvested at the same time for both developmental stages. Ten leaves (fully expanded fourth from the top) from each pot were collected for the RWC measurements^65^ and other leaf-based measurements, such as chlorophyll content (SPAD Chlorophyll Meter) and SLA as described earlier^66^. A separate set of plants were used for recording the root and shoot dry weights (after drying in draught-air oven at 65 °C till constant weight).

For salinity stress, two sets of plants of JG 62 and ICCV 2 chickpea genotypes were grown in pots. The plants were irrigated with either reverse osmosis (RO) water (control) or NaCl solution (salinity treatment) at Veg (40 mM NaCl applied before sowing and 40 mM after 8 days of sowing) and LR (two doses of 40 mM separated by 5 days at the start of flowering) stages. The roots from the control and stressed plants at both the stages were harvested after 15 days of salinity stress treatment for RNA extraction. Root tissues for all the samples were collected in at least three biological replications. Tissues were harvested, quickly wiped, frozen in liquid nitrogen and stored at −80 °C until RNA extraction. A separate set of plants (six replications) were used for various phenotypic analyses as described above for drought stress.

All the phenotypic analyses/measurements were performed in at least six replications and standard error of difference (SEd, minimum difference expected between means to be significantly different) was calculated using Genstat software.

### Illumina sequencing and data pre-processing

Total RNA was extracted from root tissues using TRI Reagent (Sigma Life Science, USA), according to manufacturer’s instructions. The quality and quantity of RNA samples (several dilutions of each) were assessed using Nanodrop Spectrophotometer (NanoDrop Technologies), Agilent Bioanalyzer (Agilent Technologies, Singapore) and agarose gel electrophoresis as described previously^67^. High-quality total RNA (RIN ≥ 8) of two biological replicates of each genotype and condition (total of 32) were processed using TrueSeq RNA Sample Prep kit (Illumina Technologies) for library preparation. The libraries were sequenced on Illumina HiSeq 2000 platform to generate more than 30 million 100 bp long paired-end (PE) reads for each sample. After multiple rounds of sequencing, we did not get sufficient coverage of high-quality reads for two samples (one replicate each of control and drought stressed samples of ICC 4958 at early reproductive stage), which resulted due to poor library yield. Therefore, finally the sequencing data from a total of 30 samples representing 16 different genotypes/conditions/developmental stage were used for further analysis. The Fastq files of raw sequence data were processed for various quality controls, including removal of low-quality reads and reads containing primer/adaptor sequences using NGS QC Toolkit (v2.3)^68^.

### Reference-guided assembly and annotation

The filtered high-quality reads were mapped on the kabuli chickpea genome (v1.0)^6^ using Tophat (v2.0.0) software with default parameters. A reference-guided assembly of the transcriptome data from all the samples was performed using Cufflinks (v2.0.2) and a consensus assembly was generated by Cuffmerge. The comparison of assembly obtained from Cuffmerge and annotated chickpea genome was done via Cuffcompare to identify novel exons/transcript isoforms and gene loci. The functional annotation of novel genes was performed via BLASTX against Arabidopsis proteome (TAIR 10) followed by SwissProt and UniProt databases.

### Identification of differentially expressed genes

The differential expression between different samples/conditions was determined by Cuffdiff. The transcripts exhibiting difference of at least two-fold change with *P*-value ≤ 0.05 were considered to be significantly differentially expressed. Log_2_-transformed FPKM values of the differentially expressed transcripts were used for K-means clustering using Pearson correlation with an optimal number of clusters to be 20 in Microarray Experiment Viewer (MeV, v4.9) software.

### GO and pathway enrichment analysis

We performed GO enrichment analysis to identify the overrepresented functional categories in the differentially expressed genes in different comparisons using BiNGO plugin of Cytoscape^69^. *P*-value for enrichment was calculated for each GO term represented and corrected via Bonferroni family-wise error rate (FWER) method. Only the GO terms exhibiting a corrected *P*-value of ≤0.05 were considered to be significantly enriched for a given set of genes. For metabolic pathway analysis, the best Arabidopsis hit of each chickpea transcript was found via BLAST search. The metabolic pathway-associated genes from AraCyc database of Plant Metabolic Network^70^ representing metabolic pathways in Arabidopsis were downloaded and pathways significantly (*P*-value ≤ 0.05) enriched in different gene sets were identified. Heatmaps representing the expression profiles of different set of transcripts were generated using MeV (http://www.tm4.org/mev.html). Pathway analysis of DETs was performed using MapMan (v3.5.1R2) software also based on the best Arabidopsis hit.

### Real-time PCR analysis

The cDNA was synthesized from 1 μg of total RNA for each sample using High-Capacity cDNA Reverse Transcription Kit (Life Technologies). The gene-specific primers (Supplementary Table S2) were designed using Primer Express (v3.0) software (Applied Biosystems, Foster City, CA). The real-time PCR analysis was performed employing ABI 7500 Real-Time PCR System (Applied Biosystems) as described previously^67^. All the reactions were performed using default parameters and specificity of the reactions was verified by melting curve analysis. The real time PCR analysis was performed with three biological replicates for each sample and three technical replicates of each biological replicate. The transcript level of each gene was normalized with the transcript level of most suitable internal control gene, *Elongation factor 1 alpha* (*EF1*α)^67^ for each sample and fold change was calculated using standard 2^−ΔΔCT^ method.

### Data availability

The entire sequencing data generated in the study have been deposited in the Gene Expression Omnibus (GEO) database under the accession numbers GSE70274 and GSE70377.

## Additional Information

**How to cite this article**: Garg, R. *et al.* Transcriptome analyses reveal genotype- and developmental stage-specific molecular responses to drought and salinity stresses in chickpea. *Sci. Rep.* **6**, 19228; doi: 10.1038/srep19228 (2016).

## Supplementary Material

Supplementary Information

## Figures and Tables

**Table 1:** Phenotypic response of contrasting chickpea genotypes ICC 4958 and ICC 1882 to drought stress.

Genotype/stress treatment	Root dry weight (g)	Total root length (cm)	Average root diameter (mm)	Shoot dry weight (g)	SLA (cm^2^/g)	Chlorophyll content	RWC
Early reproductive stage (50 day)							
ICC 4958	1.39	6535	0.820	7.96	229.0	65.3	0.639
ICC 1882	1.34	5574	0.870	5.82	268.8	62.0	0.695
SEd (±)	0.171	101.1	0.022	0.131	2.79	1.34	0.026
Significance	NS	**	**	**	**	NS	NS
Late reproductive stage (70 day)							
ICC 4958	3.85	16642	0.863	24.9	280.0	53.0	0.493
ICC 1882	3.92	18263	0.820	21.6	221.0	55.6	0.583
SEd (±)	0.291	310.7	0.023	0.483	9.85	0.42	0.029
Significance	NS	**	**	*	*	*	NS

Drought stress was imposed at the early and late reproductive stages and evaluated when the soil water remained 0.2 of the available soil water fraction in the drought stressed plants.

SLA, specific leaf area; RWC, relative water content; SEd, standard error of difference across the genotypes and stress conditions; NS, nonsignificant; *P ≤ 0.05; **P ≤ 0.01.

**Table 2:** Phenotypic response of contrasting chickpea genotypes JG 62 and ICCV 2 to salinity stress.

Genotype/stress treatment	Root dry weight (g)	Total root length (cm)	Average root diameter (mm)	Shoot dry weight (g)	SLA (cm^2^/g)	Chlorophyll content	RWC
Vegetative stage							
JG 62	0.312	1325	0.693	0.687	212.5	56.9	0.824
ICCV 2	0.294	1336	0.708	0.857	246.8	58.9	0.821
SEd (±)	0.041	480.8	0.035	0.174	12.2	1.16	0.017
Significance	NS	NS	NS	*	**	NS	NS
Late reproductive stage							
JG 62	1.18	6130	0.737	5.96	309.0	49.1	0.808
ICCV 2	0.70	3750	0.713	5.33	252.0	53.4	0.818
SEd (±)	0.283	703.1	0.070	1.96	40.4	2.13	0.018
Significance	**	**	NS	NS	*	NS	NS

Salinity stress was imposed at the time of sowing and reproductive stage and evaluated after 15 days of stress imposition.

SLA, specific leaf area; RWC, relative water content; SEd, standard error of difference across the genotypes and stress conditions; NS, nonsignificant; *P ≤ 0.05; **P ≤ 0.01.
