# Supplementary material for: Transcriptome analyses reveal genotype- and developmental stage-specific molecular responses to drought and salinity stresses in chickpea
Source: Sci Rep. 2016 Jan 13;6:19228. doi: 10.1038/srep19228 (PMC4725360; doi:10.1038/srep19228)
Supplement: Supplementary Information [file srep19228-s1.pdf]

Supplementary information

Transcriptome analyses reveal genotype- and developmental stage-specific molecular responses to drought and salinity stresses in chickpea

Rohini Garg[1], Rama Shankar[1], Bijal Thakkar[1], Himabindu Kudapa[2], Lakshmanan Krishnamurthy[2], Nitin Mantri[3], Rajeev K Varshney[2], Sabhyata Bhatia[1], Mukesh Jain[1,4]*

[1]Functional and Applied Genomics Laboratory, National Institute of Plant Genome Research (NIPGR), ArunaAsaf Ali Marg, New Delhi, India

[2]International Crops Research Institute for theSemi-Arid Tropics (ICRISAT), Patancheru, Telangana, India

[3]School of Applied Sciences, RMIT University, Victoria, Australia

[4]School of Computational & Integrative Sciences, Jawaharlal Nehru University, New Delhi, India

Supplementary Table S1 Summary of sequencing data generated, quality control and mapping on the genome.

Sample name	Genotype	Developmental stage	Condition - Replicate	Total reads	High quality reads	Mapped reads
Dtol-ER-CT1	ICC4958	Early reproductive	Control - Rep1	38946150	38357332	35132496 (91.59%)
Dtol-ER-DS1	ICC4958	Early reproductive	Drought - Rep1	87316130	86128416	79489492 (92.29%)
Dsen-ER-CT1	ICC1882	Early reproductive	Control - Rep1	90862998	89452802	80979543 (90.52%)
Dsen-ER-CT2	ICC1882	Early reproductive	Control - Rep2	56065466	54772726	50162655 (91.58%)
Dsen-ER-DS1	ICC1882	Early reproductive	Drought - Rep1	66818922	65500916	62049959 (94.73%)
Dsen-ER-DS2	ICC1882	Early reproductive	Drought - Rep2	67541626	66626432	62518842 (93.83%)
Dtol-LR-CT1	ICC4958	Late reproductive	Control - Rep1	53618608	49161672	46048054(93.66%)
Dtol-LR-CT2	ICC4958	Late reproductive	Control - Rep2	77754830	71956186	67094542(93.24%)
Dtol-LR-DS1	ICC4958	Late reproductive	Drought - Rep1	49525314	44765932	41118898(91.85%)
Dtol-LR-DS2	ICC4958	Late reproductive	Drought - Rep2	62321906	56257902	50593718(89.93%)
Dsen-LR-CT1	ICC1882	Late reproductive	Control - Rep1	72366358	67783068	64293762(94.85%)
Dsen-LR-CT2	ICC1882	Late reproductive	Control - Rep2	54605658	50106996	45508444(90.82%)
Dsen-LR-DS1	ICC1882	Late reproductive	Drought - Rep1	50837320	46399490	43714629(94.21%)
Dsen-LR-DS2	ICC1882	Late reproductive	Drought - Rep2	50934538	46921496	44210337(94.22%)
Stol-Veg-CT1	JG62	Vegetative	Control - Rep1	33828680	33268614	31723373 (95.35%)
Stol-Veg-CT2	JG62	Vegetative	Control - Rep2	40052726	39596604	33985566 (85.82%)
Stol-Veg-SS1	JG62	Vegetative	Salinity -Rep1	41280412	40823266	35693393 (87.43%)
Stol-Veg-SS2	JG62	Vegetative	Salinity -Rep2	33665672	33046724	31259781 (94.59%)
Ssen-Veg-CT1	ICCV2	Vegetative	Control - Rep1	34060046	33544722	31148113 (92.85%)
Ssen-Veg-CT2	ICCV2	Vegetative	Control - Rep2	32694562	31906180	30280027 (94.90%)
Ssen-Veg-SS1	ICCV2	Vegetative	Salinity -Rep1	32702022	32176314	30383497 (94.42%)
Ssen-Veg-SS2	ICCV2	Vegetative	Salinity -Rep2	33709024	32875038	26330836 (80.09%)
Stol-LR-CT1	JG62	Late reproductive	Control - Rep1	53802474	44144472	40383552(91.48%)
Stol-LR-CT2	JG62	Late reproductive	Control - Rep2	43672212	36478766	33420893(91.61%)
Stol-LR-SS1	JG62	Late reproductive	Salinity -Rep1	34373030	30962394	28850367 (93.17%)
Stol-LR-SS2	JG62	Late reproductive	Salinity -Rep2	48326362	39824296	35768344(89.81%)
Ssen-LR-CT1	ICCV2	Late reproductive	Control - Rep1	43921582	38496050	35515817(92.25%)

Ssen-LR-CT2	ICCV2	Late reproductive	Control - Rep2	46497994	41874556	37609076(89.81%)
Ssen-LR-SS1	ICCV2	Late reproductive	Salinity -Rep1	40374538	34562396	31796256(91.99%)
Ssen-LR-SS2	ICCV2	Late reproductive	Salinity -Rep2	41832144	36349044	33460675(92.05%)

Supplementary Table S2. List of primers sequences used for real time PCR analysis.

Gene ID	Primer sequence
Ca_02451	GATTCAACCAAAGAGACTCCAACA
	GATAACCCTTTTTGTAGCTTTTATTGTCA
Ca_23716	TGAAAATGATGCTGTGAGTACTAGGA
	TGTCCCAGAAGATACCAGAGGAAT
Ca_08236	CTGATGCACCAGCTCCAAGTC
	GAGAAGCAAAAGCAGTTGGAACA
Ca_03790	GCTGAGATTTTTGAGGCGGTTA
	AGATCGGAAGATTCTGGATGGA
Ca_14421	CGAACGACCACATCGGAATC
	GGCTCCGGTAAGTGATGTAGGT
Ca_05907	TCGCACAAATCCAACCATACA
	CGCCGTTGAATCGCTCAT
Ca_11127	GCCACCGCTTGTGATCGT
	GAGCAGAAACTTTGGAGAAATAGGAA
Ca_16693	AAAGAACGAGGACCCGTGATAA
	GCAGGAGGAATACCCGACAA
Ca_24924	TGATGATGTTGATTACCTTGTTGTTG
	TCTCTCCAATTCCTTAGCAGCAT

Supplementary Fig. S1 Venn diagrams showing overlap between the number of genes differentially expressed in the chickpea roots at the seedling (previous study) and ER/LR/Veg stages (present study) under drought (a,b) and salinity (c,d) stress. Root-DS and Root-SS represent number of differentially expressed genes in the roots of chickpea seedlings under drought and salinity stress, respectively (from Garg et al., 2015, Plant Mol Biol Rep). The number of unique gene loci showing differential expression in the roots at ER/LR/Veg stage under drought/salinity stress for other samples from the present study are represented.

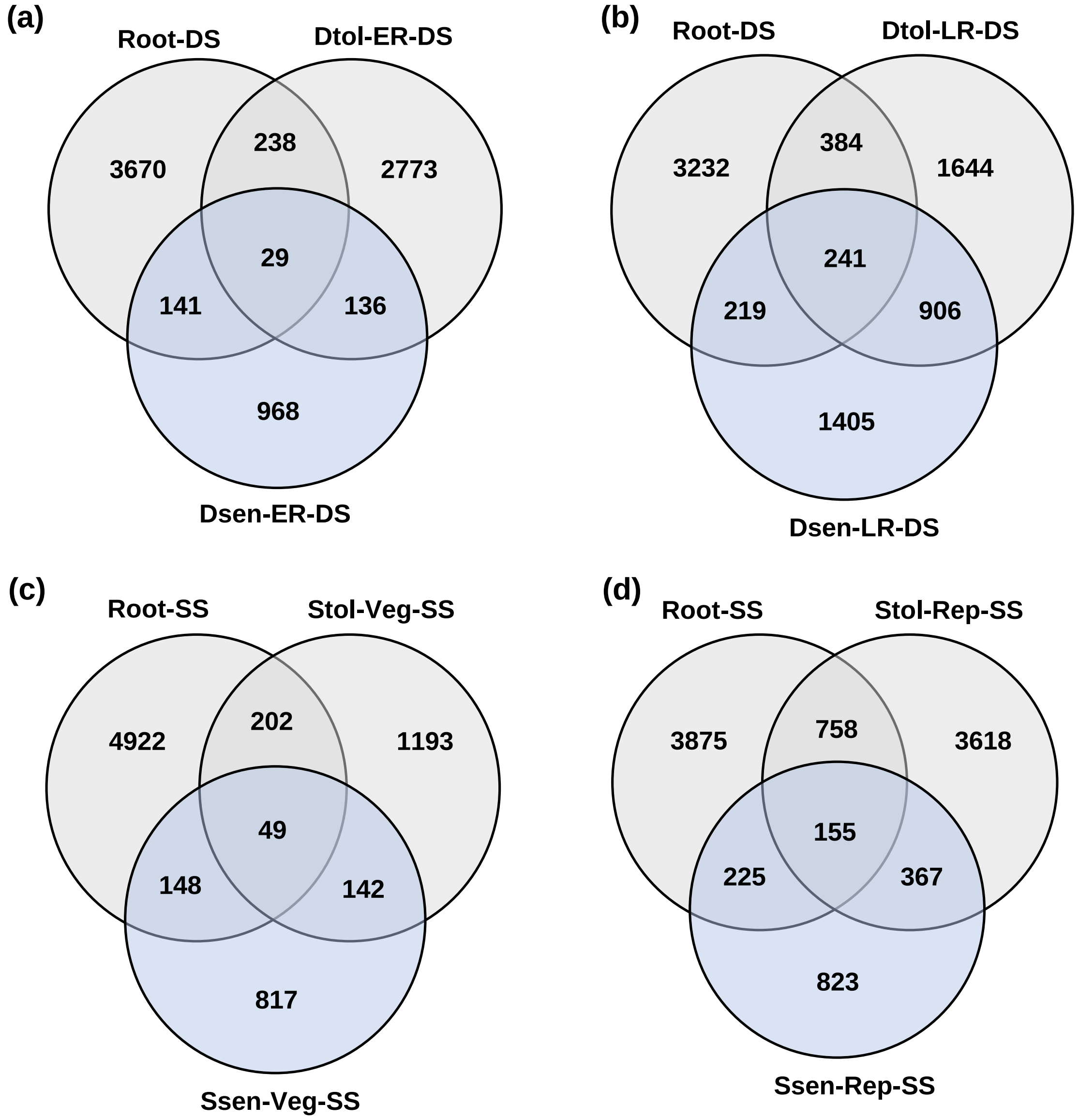

Supplementary Fig. S2 Heatmaps showing the differential expression of transcripts involved in stomatal regulation (a) and ion homeostasis (b). Color scale showing differential expression (log_2 fold change) is shown at the bottom.The related biological process GO terms are shown on the right side.

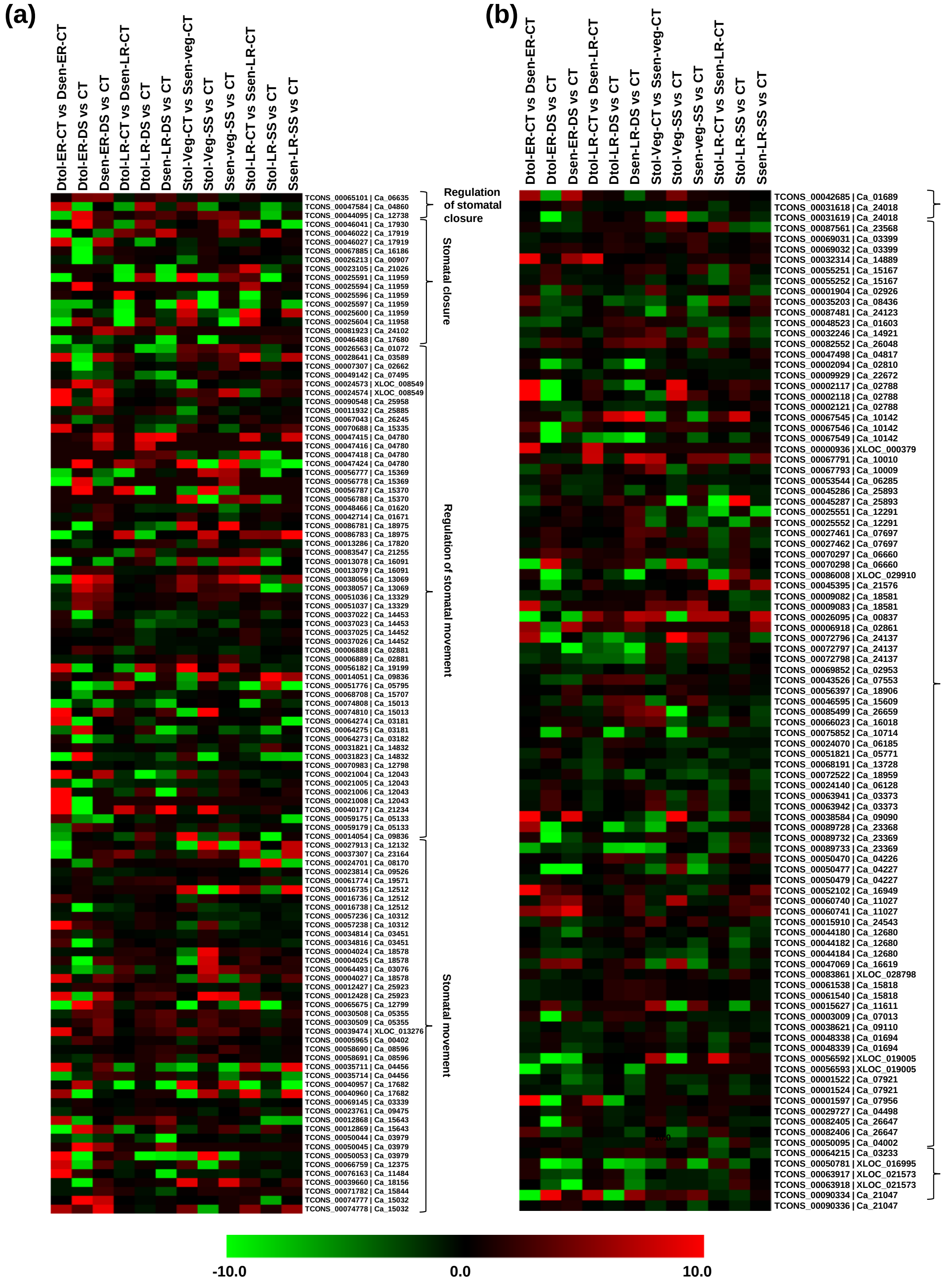

Supplementary Fig. S3 Quantitative real-time PCR analysis to validate the results of RNA-seq. The bar graph shows relative transcript abundance of different chickpea genes in different comparisons (labeled below the lower panel) obtained via RT-qPCR and RNA-seq analyses. 'r' represents correlation coefficient between RNA-seq and RT-qPCR data.

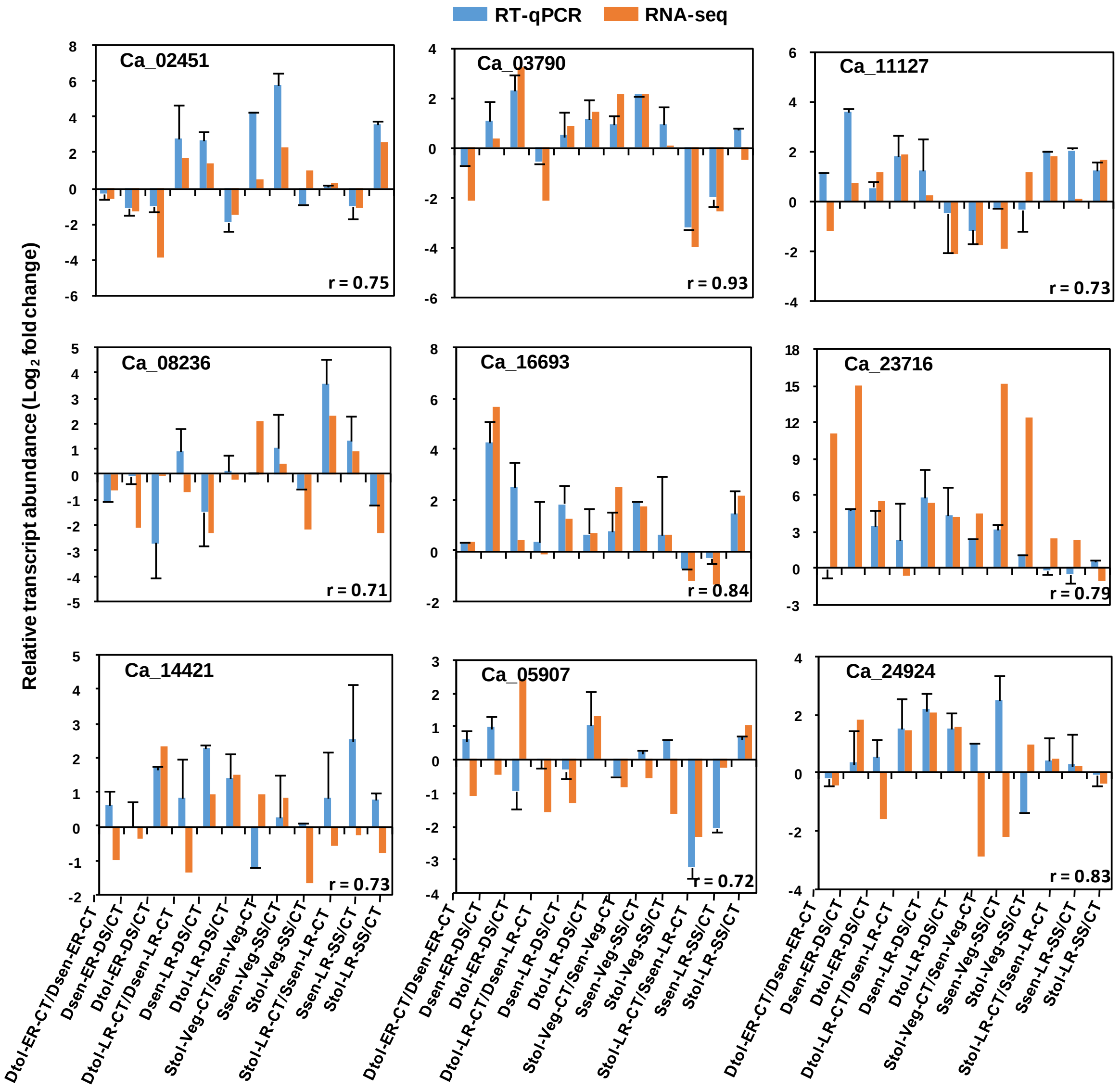

Supplementary Fig. S4 Number of transcripts and gene loci representing AP2-EREBP family members showing differential expression under different conditions.

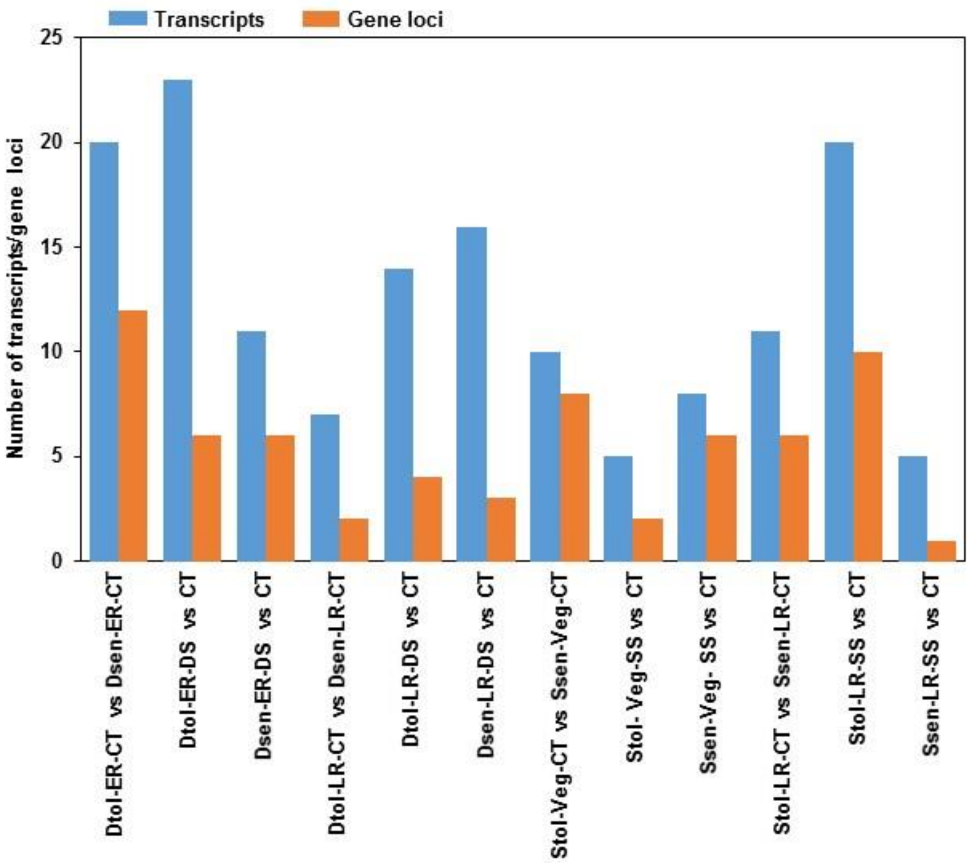

Supplementary Fig. S5 Metabolic pathway overview of the differentially expressed transcripts in drought related cultivars obtained from MapMan analysis. The differential expression of genes involved in different metabolic pathways is shown in the form of heatmaps. Color scale showing fold change is shown at the bottom. Metabolic pathways are represented in different shapes of grey color.

Dsen-ER-DS/CT

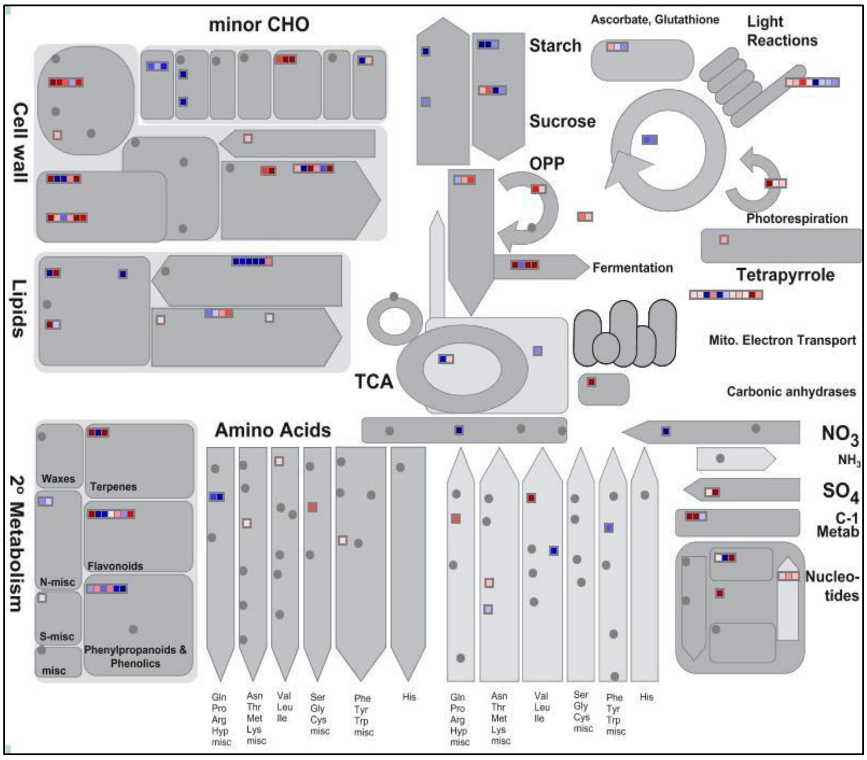

Dsen-LR-DS/CT

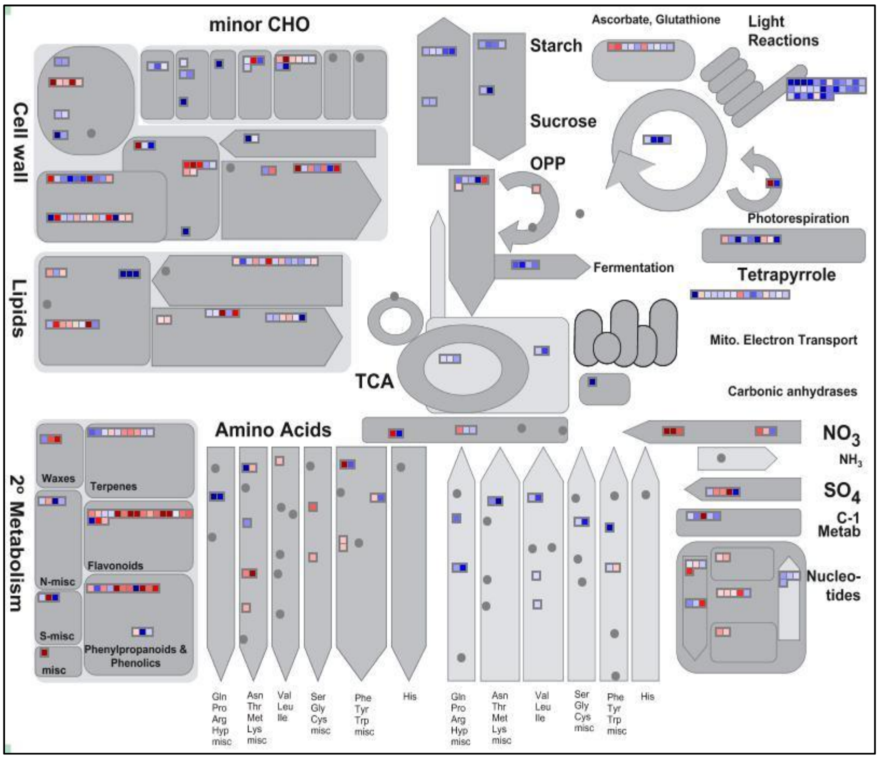

DtoI-ER-DS/CT

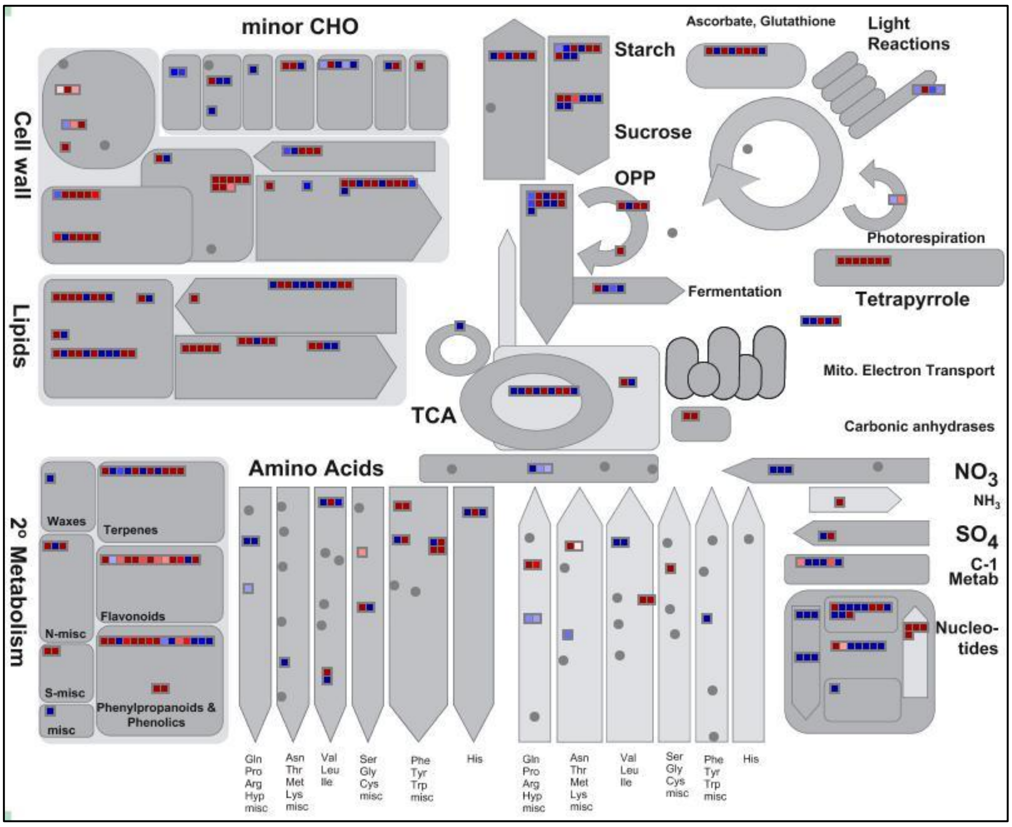

DtoI-LR-DS/CT

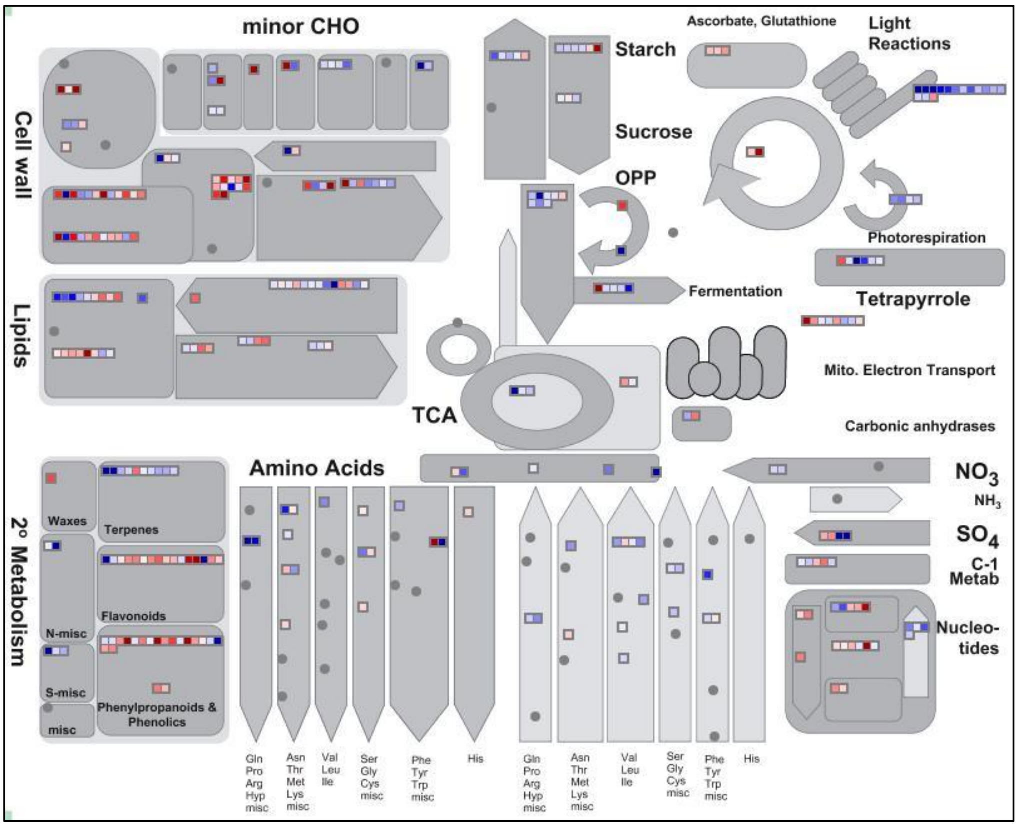

Dtol-ER-CT/Dsen-ER-CT

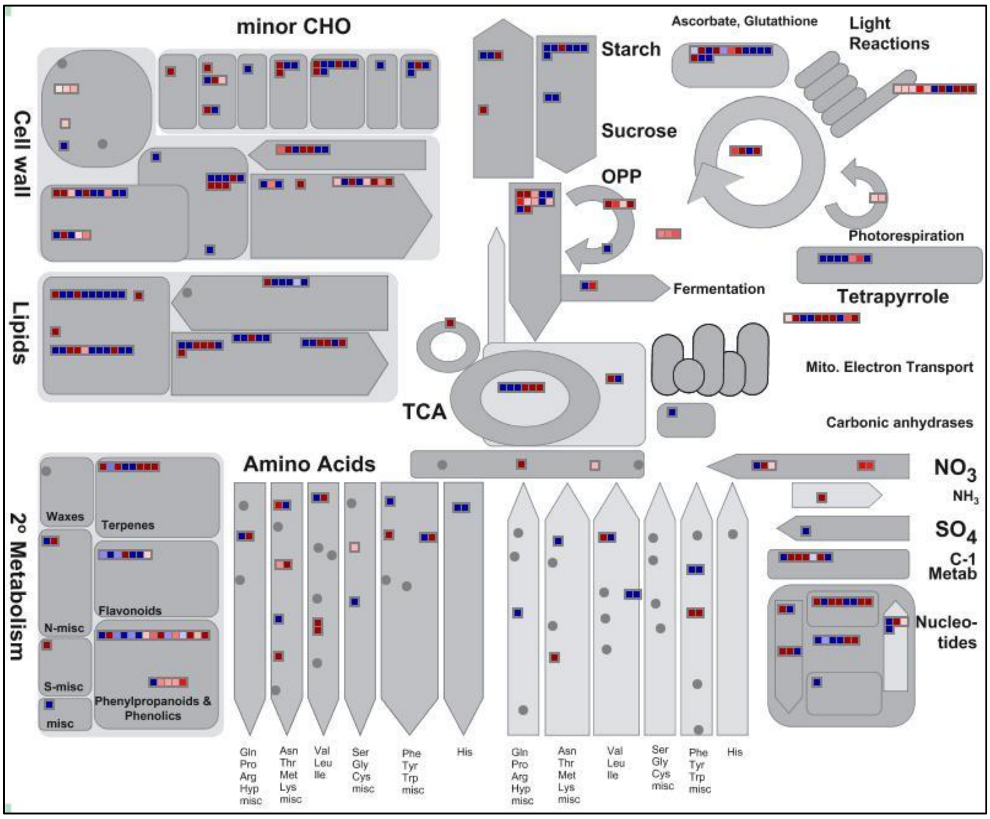

Dtol-LR-CT/ Dsen-LR-CT

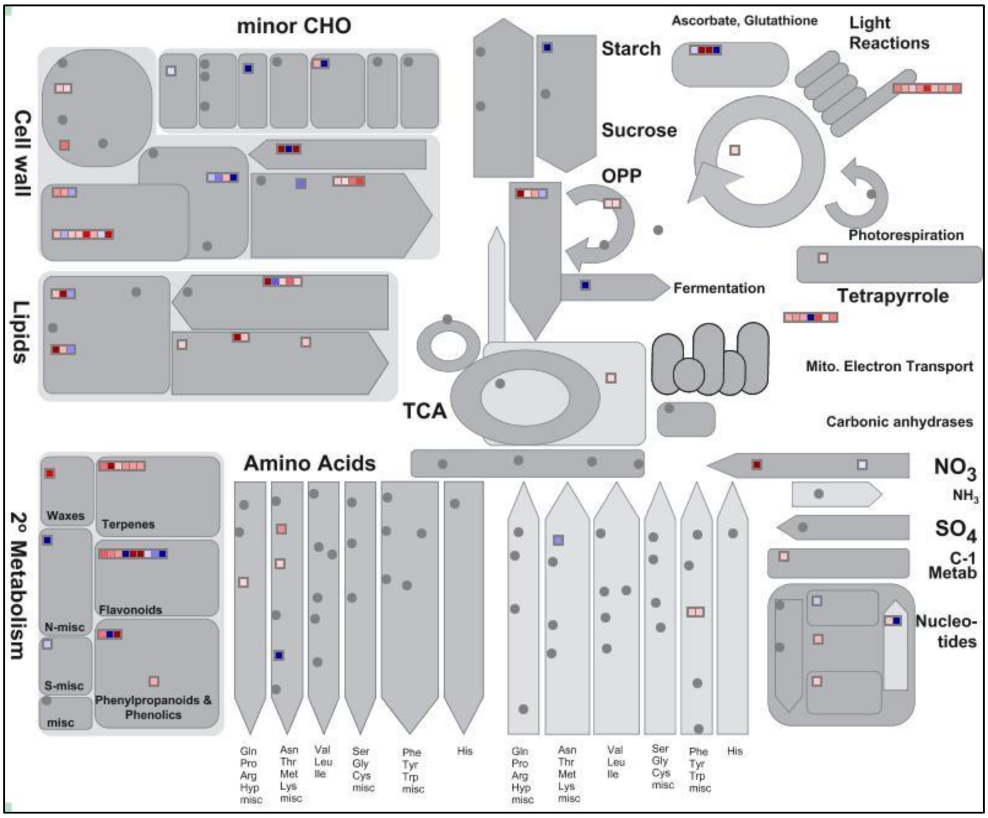

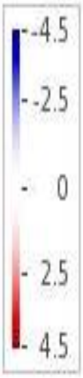

Supplementary Fig. S6 Metabolic pathway overview of the differentially expressed transcripts in salinity related cultivars obtained from MapMan analysis. The differential expression of genes involved in different metabolic pathways is shown in the form of heatmaps. Color scale showing fold change is shown at the bottom. Metabolic pathways are represented in different shapes of grey color.

Ssen-Veg-SS/CT

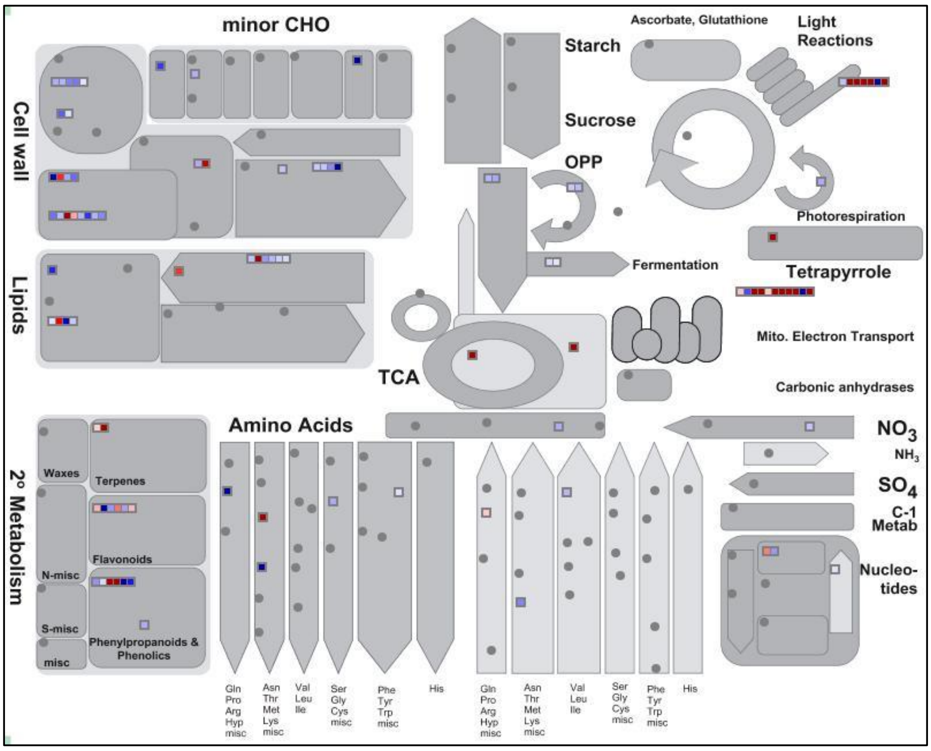

Ssen-LR-SS/CT

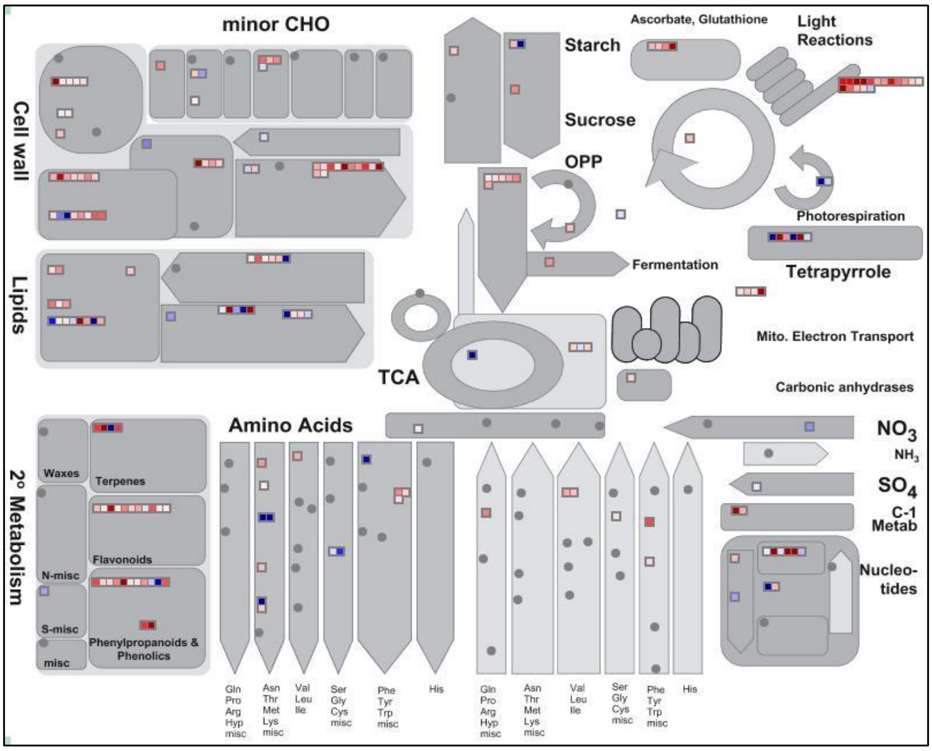

Stol-Veg-SS/CT

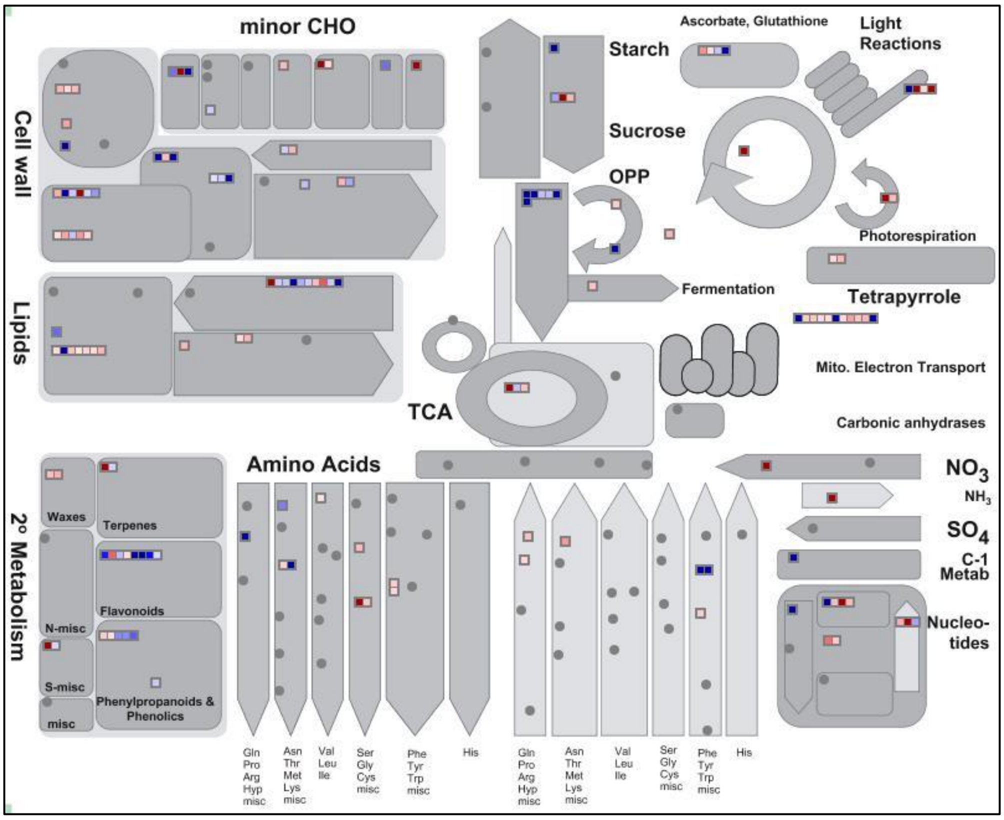

Stol-LR-SS/CT

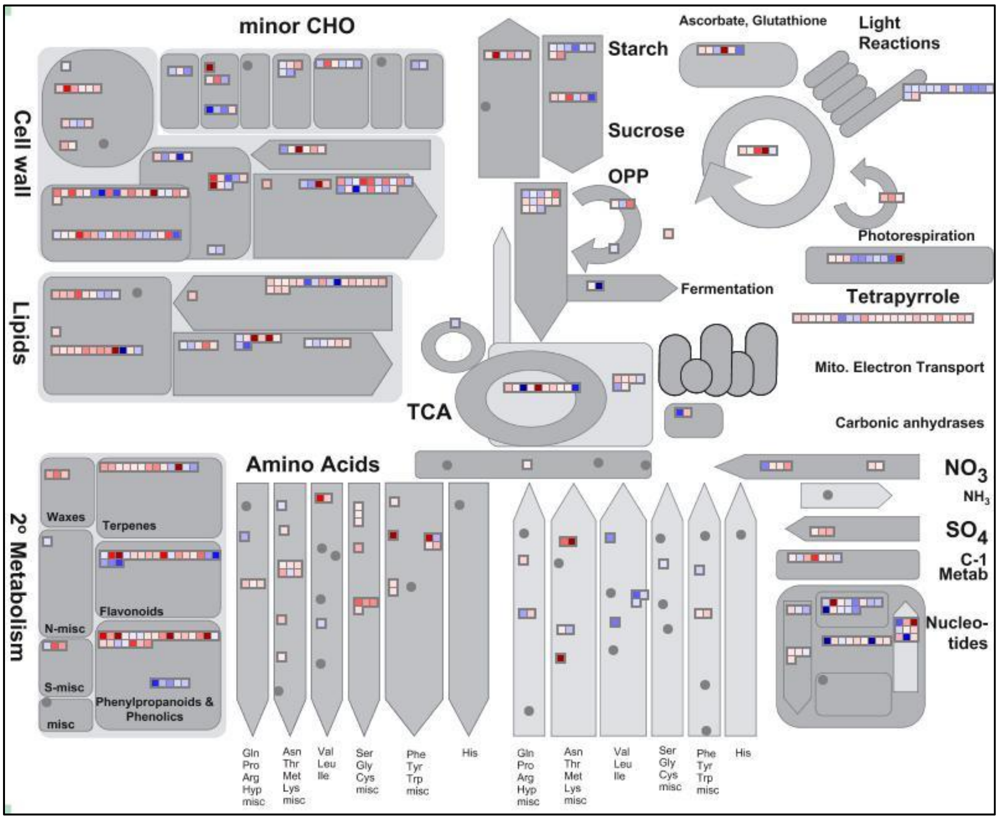

Stol-Veg-CT/Ssen-Veg-CT

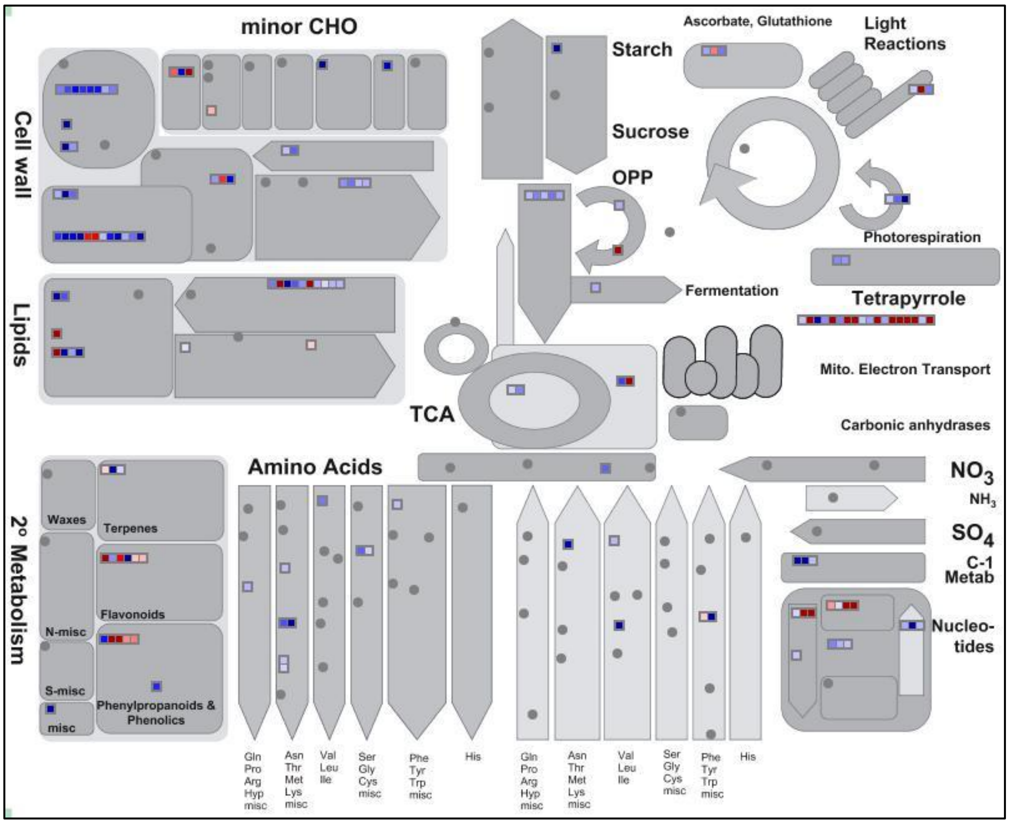

Stol-LR-CT/ Ssen-LR-CT

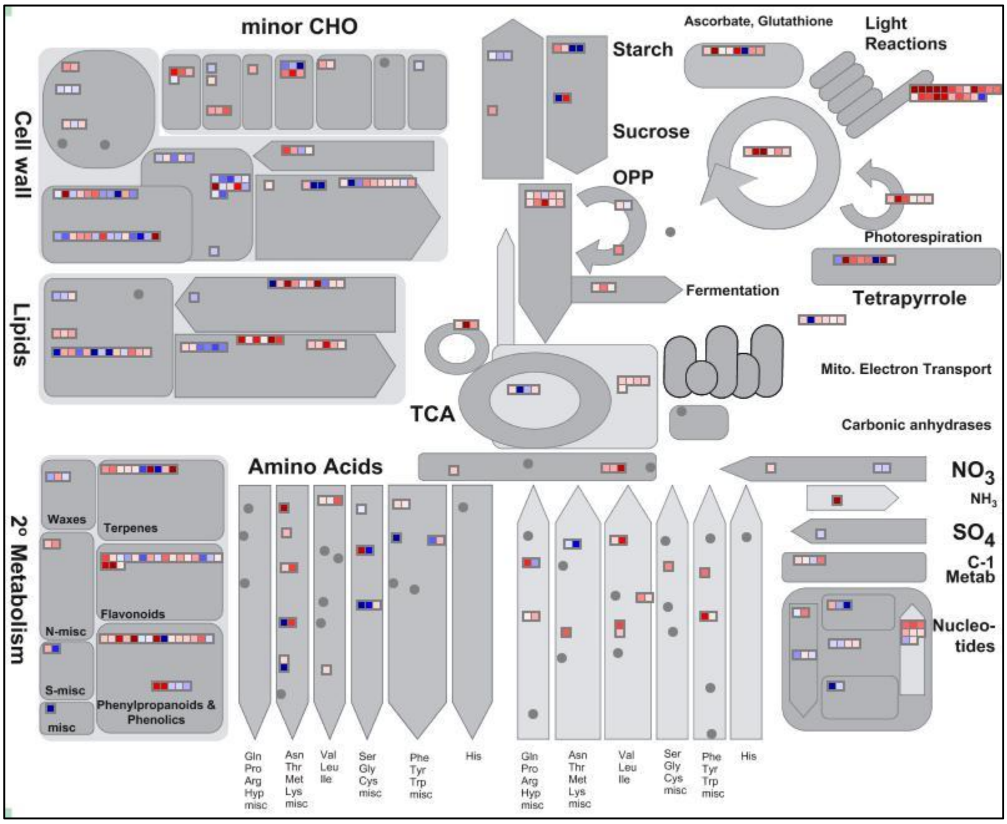

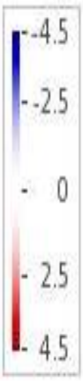

Supplementary Fig. S7 Biotic stress pathway overview of the differentially expressed transcripts in drought-tolerant cultivars obtained from MapMan analysis. The differential expression of genes involved in different biotic stress related pathways is shown in the form of heatmaps. Color scale showing differential expression is shown at the top left.

Dtol-ER-CT/Dsen-ER-CT

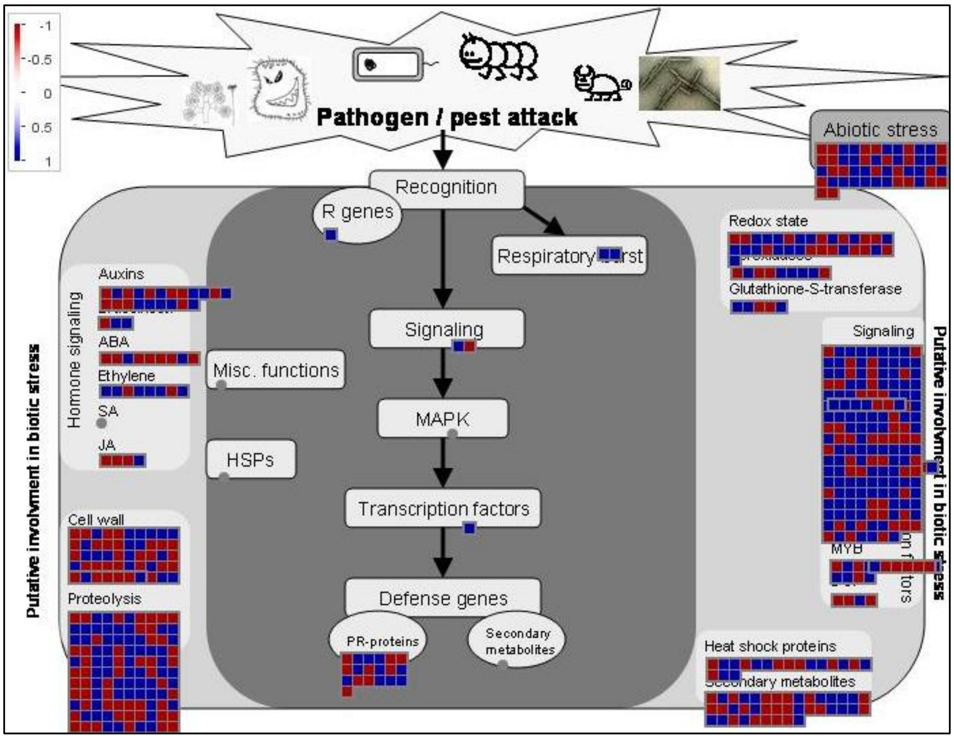

Dtol-ER-DS/CT

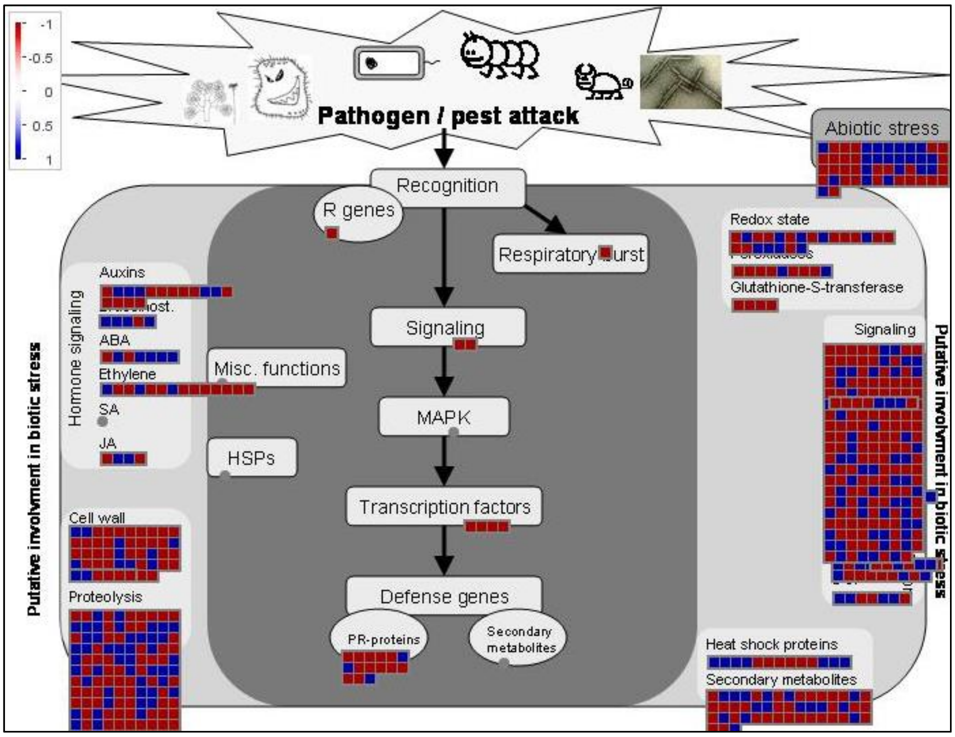

The above images are the output from the MapMan software. The pathogen/pest images and pathways represented are the standard output of the MapMan for biotic stress pathway and heatmaps are drawn by the MapMan software accroding to the differnetially expressed transcripts involved in these pathways.

Supplemental Fig. S8 Biotic stress pathway overview of the differentially expressed transcripts in salinity-tolerant cultivars obtained from MapMan analysis. The differential expression of genes involved in different biotic stress related pathways is shown in the form of heatmaps. Color scale showing differential expression is shown at the top left.

Stol-LR-CT/ Ssen-LR-CT

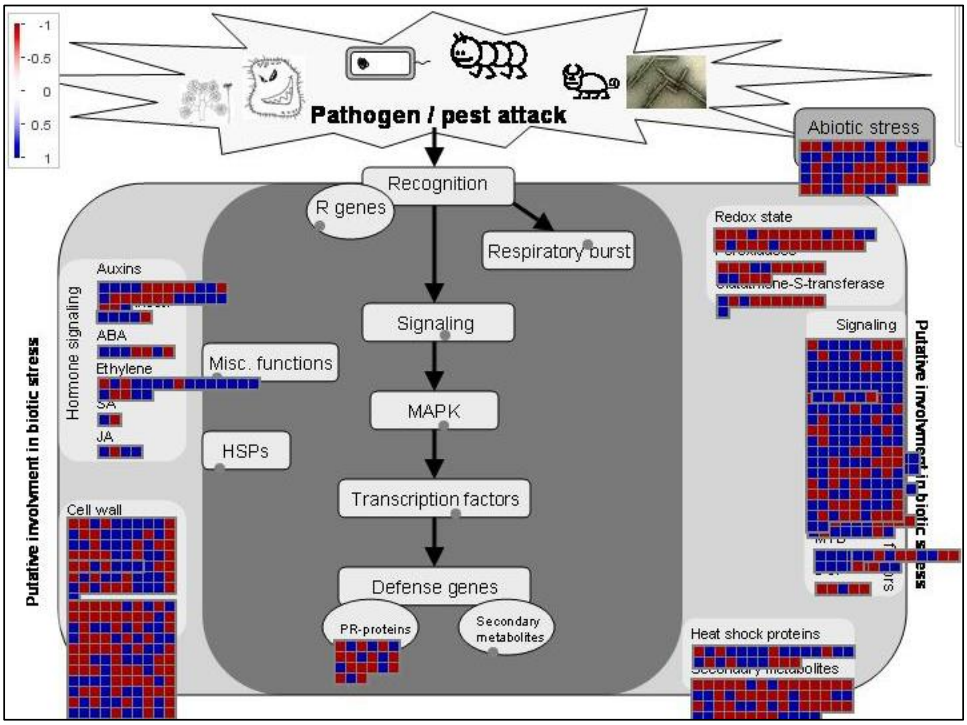

Stol-LR-SS/CT

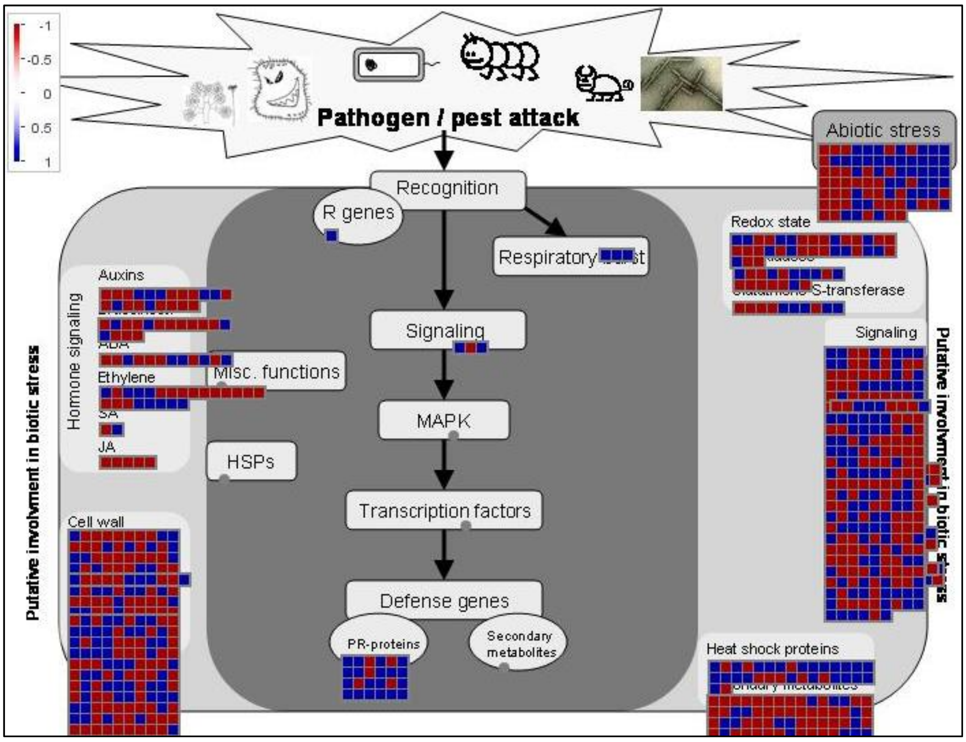

The above images are the output from the MapMan software. The pathogen/pest images and pathways represented are the standard output of the MapMan for biotic stress pathway and heatmaps are drawn by the MapMan software accroding to the differnetially expressed transcripts involved in these pathways.
